# The HILDA Complex Coordinates a Conditional Switch in the 3′-Untranslated Region of the *VEGFA* mRNA

**DOI:** 10.1371/journal.pbio.1001635

**Published:** 2013-08-20

**Authors:** Peng Yao, Alka A. Potdar, Partho Sarothi Ray, Sandeepa M. Eswarappa, Andrew C. Flagg, Belinda Willard, Paul L. Fox

**Affiliations:** 1 Department of Cellular and Molecular Medicine, Lerner Research Institute, Cleveland Clinic, Cleveland, Ohio, United States of America; 2 Department of Biomedical Engineering, Case Western Reserve University, Cleveland, Ohio, United States of America; 3 Department of Biology, Indian Institute of Science Education and Research, Kolkata, India; 4 Mass Spectrometry Laboratory for Protein Sequencing, Lerner Research Institute, Cleveland Clinic, Cleveland, Ohio, United States of America; Brigham and Women's Hospital Harvard School of Medicine, United States of America

## Abstract

Cell regulatory circuits integrate diverse, and sometimes conflicting, environmental cues to generate appropriate, condition-dependent responses. Here, we elucidate the components and mechanisms driving a protein-directed RNA switch in the 3′UTR of vascular endothelial growth factor (VEGF)-A. We describe a novel HILDA (hypoxia-inducible hnRNP *L*–*D*RBP76–hnRNP *A*2/B1) complex that coordinates a three-element RNA switch, enabling *VEGFA* mRNA translation during combined hypoxia and inflammation. In addition to binding the CA-rich element (CARE), heterogeneous nuclear ribonucleoprotein (hnRNP) L regulates switch assembly and function. hnRNP L undergoes two previously unrecognized, condition-dependent posttranslational modifications: IFN-γ induces prolyl hydroxylation and von Hippel-Lindau (VHL)-mediated proteasomal degradation, whereas hypoxia stimulates hnRNP L phosphorylation at Tyr^359^, inducing binding to hnRNP A2/B1, which stabilizes the protein. Also, phospho-hnRNP L recruits DRBP76 (double-stranded RNA binding protein 76) to the 3′UTR, where it binds an adjacent AU-rich stem-loop (AUSL) element, “flipping” the RNA switch by disrupting the GAIT (interferon-gamma-activated inhibitor of translation) element, preventing GAIT complex binding, and driving robust *VEGFA* mRNA translation. The signal-dependent, HILDA complex coordinates the function of a trio of neighboring RNA elements, thereby regulating translation of *VEGFA* and potentially other mRNA targets. The *VEGFA* RNA switch might function to ensure appropriate angiogenesis and tissue oxygenation during conflicting signals from combined inflammation and hypoxia. We propose the *VEGFA* RNA switch as an archetype for signal-activated, protein-directed, multi-element RNA switches that regulate posttranscriptional gene expression in complex environments.

## Abstract 2

The HILDA complex coordinates three regulatory elements located in the 3′ UTR of the *VEGFA* mRNA in a RNA switch that controls translation in response to inflammation and hypoxia.

## Author Summary

Many cells of our body, particularly cells such as monocyte/macrophages involved in host immunity, are exposed to diverse and constantly changing environments. These cells require mechanisms by which they can rapidly respond to multiple, sometimes conflicting, environmental cues to generate appropriate responses. The 3′ untranslated regions (UTRs), i.e. the noncoding tail of messenger RNAs, often contain multiple protein- and RNA-binding elements, thereby making it an ideal setting for receiving multiple such environmental cues, which can then be integrated into a single response that regulates the gene's expression. Monocytic cells exposed to inflammation and hypoxia produce vascular endothelial growth factor (VEGF)-A, a critical factor in blood vessel formation. VEGF-A expression is regulated under these conditions via a complex regulatory mechanism that involves its 3′UTR. Here we show how this regulatory switch works. Inflammation induces formation of a four-protein complex that binds an RNA element present in the *VEGFA* 3′UTR and blocks translation. Hypoxia, however, triggers the assembly of a completely different three-protein complex (termed “HILDA”) that coordinates the function of three neighboring RNA elements to “flip” the RNA conformation in such a way that prevents the first complex from binding, thereby allowing VEGF-A expression. We hypothesize that the *VEGFA* switch might function to ensure appropriate angiogenesis and tissue oxygenation when cells are exposed to conflicting signals from combined inflammation and hypoxia conditions.

## Introduction

Mammalian cells integrate diverse, and sometimes conflicting, environmental signals to generate appropriate, condition-dependent responses. Tissue myeloid cells are exposed to a plethora of stimulatory and inhibitory signals, and thus its integrated response is particularly complex. This task is made more problematical, and possibly more critical, in dynamic, pathological environments. Myeloid cell vascular endothelial growth factor (VEGF)-A is critical for blood vessel formation during development, wound-healing, and tumorigenesis [1]. Hypoxia is possibly the most potent agonist of VEGF-A expression, working at the levels of transcription, mRNA stabilization, and translation [2],[3]. VEGF-A synthesis is induced in monocyte/macrophages activated by pro-inflammatory agonists, including interferon (IFN)-γ and bacterial lipopolysaccharide. Overproduction of VEGF-A can cause excessive neovascularization, blood vessel permeability, and enhanced leukocyte recruitment, all hallmarks of chronic inflammatory conditions, including cancer and atherosclerosis [4]–[6]. Agents that inhibit VEGF-A or its receptor have been applied clinically to successfully limit colorectal and renal cell carcinoma [7]. Positive and negative regulation of VEGF-A expression has been reported in human macrophages in multiple stressed conditions. We have shown that VEGF-A expression in myeloid cells is translationally repressed by the IFN-γ-triggered GAIT (interferon-gamma-activated inhibitor of translation) system [8],[9]. Importantly, under certain pathological conditions, for example within the avascular cores of tumors and in the thickened intima of atherosclerotic lesions, macrophages are simultaneously exposed to both inflammatory cytokines and hypoxia that act concurrently in multiple pathophysiological scenarios to regulate gene expression.

Treatment of human monocytic cells with IFN-γ induces the synthesis of *VEGFA* mRNA and protein for up to about 12 to 16 h. However, VEGF-A synthesis and secretion are suppressed about 16 h after IFN-γ treatment despite the presence of abundant *VEGFA* mRNA [10]. Translational silencing of *VEGFA* and other GAIT targets requires binding of the GAIT complex to its cognate GAIT element in the target mRNA 3′UTR [10]. The GAIT element is a defined 29-nt stem-loop with an internal bulge and unique sequence and structural features. The human GAIT complex is heterotetrameric containing glutamyl-prolyl-tRNA synthetase (EPRS), ribosomal protein L13a, NS1-associated protein–1, and glyceraldehyde 3-phosphate dehydrogenase (GAPDH) [11],[12]. A C-terminus truncated form of EPRS, termed EPRS^N1^, functions as a dominant-negative regulator of GAIT complex activity and maintains basal expression of VEGF-A [13].

RNA-binding proteins (RBPs) that regulate mRNA stability or translation generally recognize their target mRNAs through structural or sequence-specific elements in the 5′ or 3′UTRs of mature mRNAs. The activity of trans-acting RBPs can be modulated by dosage (in turn regulated by synthesis rate and stability), cellular localization, posttranslational modification, noncoding RNAs, and interacting protein partners. Heteronuclear ribonucleoprotein (hnRNP) L is a key posttranscriptional regulator of VEGF-A expression. Human hnRNP L has three consensus RNA recognition motifs (RRM) [14] and binds CA-rich elements (CARE) in coding and noncoding regions of multiple transcripts [15]. hnRNP L contributes to pre-mRNA splicing [16], mRNA nucleocytoplasmic transport [14], internal ribosomal entry site-mediated translation [17], translational repression [18], and mRNA stabilization [19].

The molecular mechanisms by which signal transduction systems integrate multiple environmental cues into a binary response that determines gene expression remain largely unexplored. We have reported that hnRNP L operates a hypoxia-stimulated, binary conformational RNA switch that overrides IFN-γ-induced GAIT-mediated translational silencing of *VEGFA* mRNA in human monocytic U937 cells and in primary human peripheral blood monocytes (PBMs) [20]. The proposed switch permits high-level VEGF-A expression under combined inflammatory and hypoxic stress. Here we elucidate the molecular mechanism underlying the IFN-γ- and hypoxia-dependent regulatory RNA switch. The switching mechanism involves condition-dependent posttranslational modification and relocalization of hnRNP L, and subsequent formation of an hnRNP L-containing heterotrimeric complex that stabilizes the *VEGFA* HSR in a translation-competent conformation.

## Results

### VEGFA RNA Switch Is a Heterotrimeric Complex Containing hnRNP L, DRBP76, and hnRNP A2/B1

**Figure 1:**
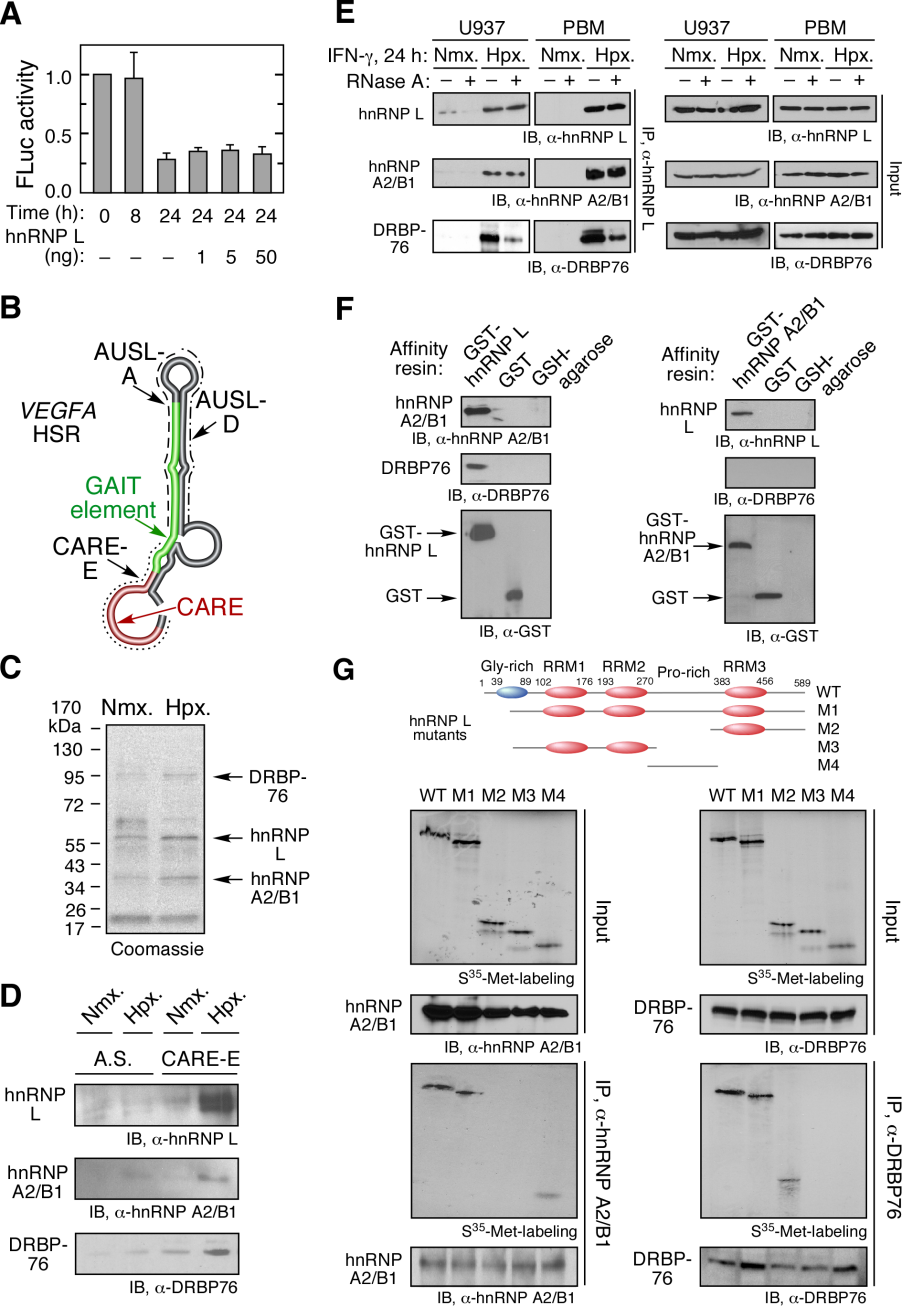
Heterotrimeric HILDA complex binds the *VEGFA* HSR in hypoxia. (A) Recombinant hnRNP L by itself does not drive the *VEGFA* RNA switch and restore *in vitro* translation of the GAIT-element-bearing reporter. *In vitro* translation of capped and poly(A)-tailed firefly luciferase (*FLuc*)-*VEGFA* HSR-A_30_ reporter transcript was determined in a wheat germ extract in the presence of [^35^S]Met, cytosolic extracts from IFN-γ-treated U937 cells, and recombinant hnRNP L. FLuc expression was determined by activity assay, normalized by RLuc expression, and reported as mean ± standard deviation (SD, *n* = 3). (B) Schematic of HSR in *VEGFA* 3′UTR. CARE (red), GAIT element (green), extended CARE (CARE-E, dotted line), AUSL-A (dashed line), and AUSL-D (dashed and dotted line) are indicated. (C) Mass spectrometric analysis of CARE-binding proteins. U937 cells were treated with normoxia (Nmx.) or hypoxia (Hpx.) for 24 h and the S100 extracts, precleared, and incubated with biotinylated CARE-E (extended CARE, sequences in Materials and Methods) and magnetic streptavidin microbeads. Specifically bound proteins were subjected to SDS-PAGE and Coomassie staining. Bands specifically enriched in affinity-purified lysates from hypoxia-treated cells were trypsinized, and peptide sequences of hnRNP L, DRBP76, and hnRNP A2/B1 detected by mass spectrometry. (D) Hypoxia-inducible binding of hnRNP L, DRBP76, and hnRNP A2/B1 to CARE. Cells were treated with Nmx. or Hpx. for 24 h, and the precleared S100 extracts incubated with biotinylated, wild-type, or antisense (A.S.) CARE-E, and then with magnetic streptavidin microbeads. Specifically bound proteins were subjected to immunoblot analysis. (E) DRBP76 and hnRNP A2/B1 form a complex with hnRNP L *in vivo*. Cells were treated with IFN-γ in Nmx. or Hpx. for 24 h. Cell lysates were incubated with or without RNase A, immunoprecipitated with anti-hnRNP L antibody, and subjected to immunoblot analysis (left panel). Total expression of hnRNP L, hnRNP A2/B1, and DRBP76 was determined by immunoblot as input control (right panel). (F) Interprotein interactions of HILDA constituents. Recombinant hnRNP A2/B1 and DRBP76 were incubated with GST-hnRNP L or GST immobilized to glutathione (GSH)-agarose beads. After washing, binding was detected by immunoblot (left). Recombinant hnRNP L and DRBP76 were incubated with GST-hnRNP A2/B1 or GST immobilized to GSH-agarose beads (right). (G) hnRNP L domain mapping. *In vitro* synthesized S^35^-Met-labeled hnRNP L segments (top) were incubated with cytosol from U937 cells. hnRNP A2/B1 (left) and DRBP76 (right) were immunoprecipitated, and the interacting hnRNP L segments detected by autoradiorgraphy. Key hnRNP L domains are shown above.

HnRNP L is an essential component of the RNA switch that blocks GAIT-mediated translational silencing of *VEGF-A* mRNA, and permits high-level expression of VEGF-A in myeloid cells in the presence of IFN-γ and hypoxia (Figure S1) [20]. To determine whether hnRNP L is sufficient for RNA switch function, the activity of recombinant protein was determined by *in vitro* translation of luciferase reporter bearing the *VEGFA* HSR in a wheat germ extract system in the presence of active GAIT complex from IFN-γ-treated U937 cells (Figure 1A). hnRNP L failed to overcome the translational repression suggesting that posttranslational modification of hnRNP L or additional protein factors may be required. Identical results were seen using a rabbit reticulocyte lysate system (not shown). Hypoxia-dependent hnRNP L binding partners were determined by RNA affinity purification using a 30-nt, 5′-biotinylated, extended CARE (CARE-E) from the *VEGFA* HSR (Figure 1B). To reduce nonspecific binding, lysates from U937 cells incubated under normoxic or hypoxic conditions were pre-cleared with an excess of 5′-biotinylated antisense CARE-E RNA in which CA pairs were mutated to GU. Cleared lysates were incubated with biotinylated, wild-type CARE-E RNA and μMAC magnetic streptavidin microbeads, and applied to a magnetic column. Bound proteins were eluted with salt solution, concentrated, and subjected to SDS-PAGE and Coomassie stain (Figure 1C). Bands enriched in lysates from hypoxia-treated cells were subjected to mass spectrometric analysis, and peptides corresponding to hnRNP L, hnRNP A2/B1, and DRBP76 (nuclear factor 90 or interleukin enhancer binding factor 3) were identified (Table S1). Binding of the proteins to CARE RNA was confirmed by RNA affinity isolation and immunoblot analysis of lysates from hypoxia-treated U937 cells. A hypoxia-inducible complex of hnRNP L, DRBP76, and hnRNP A2/B1 (HILDA) was shown to bind wild-type but not mutant antisense CARE RNA; substantially less binding of the three proteins to CARE RNA was observed in normoxic lysates (Figure 1D).

The formation of an RNA-binding heterotrimeric complex was investigated by co-immunoprecipitation (IP). Lysates from U937 cells and primary human PBM treated with IFN-γ under normoxic or hypoxic conditions were subjected to IP with anti-hnRNP L antibody, and probed with hnRNP A2/B1- and DRBP76-specific antibodies (Figure 1E, left panel). A hypoxia-dependent interaction of hnRNP L with hnRNP A2/B1 and DRBP76 was observed. The interaction between hnRNP L and hnRNP A2/B1 was RNA-independent as shown by the lack of an effect of RNase A treatment. However, the RNase diminished the interaction between hnRNP L and DRBP76, suggesting that the hnRNP L-DRBP76 complex is stabilized by RNA. The expression levels of the three HILDA complex constituents were not altered by hypoxia exposure (Figure 1E, right panel). *In vitro* GST-pulldown experiments showed that recombinant GST-hnRNP L directly interacted with recombinant hnRNP A2/B1 and DRBP76 (Figure 1F, left panel). In a parallel experiment, GST-hnRNP A2/B1 was found to directly bind hnRNP L but not DRBP76 (Figure 1F, right panel). hnRNP L contains an N-terminal glycine-rich domain, three RNA-binding motifs (RRM1–3), and a proline-rich linker domain connecting RRM2 and RRM3 (Figure 1G, top). Domain mapping experiments revealed that hnRNP A2/B1 binds the proline-rich linker in hnRNP L (Figure 1G, left). In contrast, the RRM3-containing, C-terminal domain of hnRNP L was the binding site for DRBP76 (Figure 1G, right).

**Figure 2:**
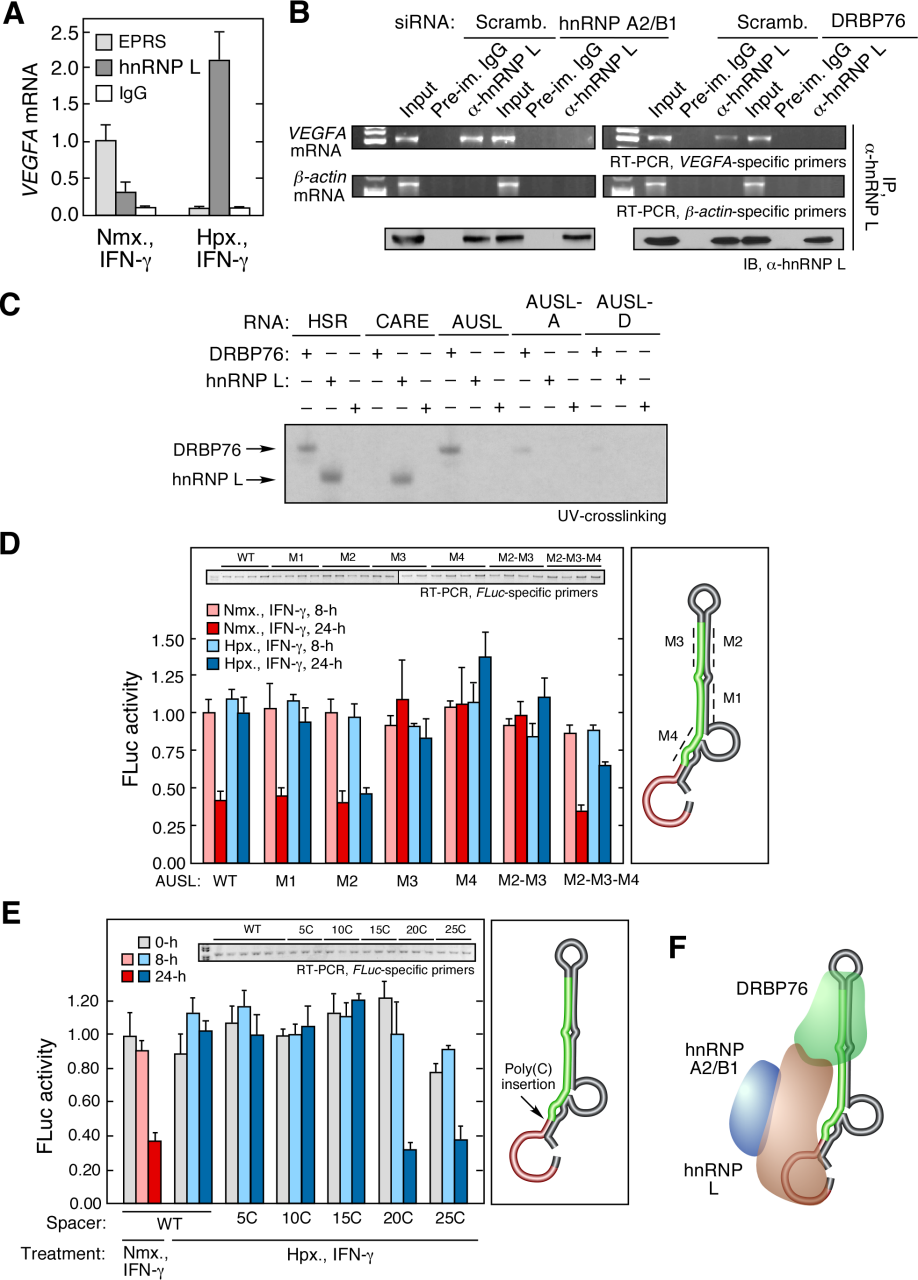
Specific HSR RNA requirements for DRBP76 interaction and RNA switch activity. (A) Condition-dependent binding of *VEGFA* mRNA to GAIT and HILDA complexes. U937 cells were treated with IFN-γ for 24 h under Nmx. or Hpx. Lysates were subjected to IP with anti-hnRNP L or -EPRS antibodies (or IgG control) coupled with qRT-PCR using gene-specific primers. *VEGFA* mRNA was normalized to *GAPDH* mRNA, and results normalized to amount of *VEGFA* mRNA in EPRS IP of cells treated with IFN-γ under Nmx. (B) hnRNP A2/B1 and DRBP76 are essential for hnRNP L binding to *VEGFA* mRNA *in vivo*. U937 cells were transfected with hnRNP A2/B1- and DRBP76-specific (or scrambled) siRNA, and lysates immunoprecipitated with anti-hnRNP L antibody. Extracted RNA was subjected to RT-PCR using primers specific for *VEGFA* or *β-actin* mRNA. Efficiency of hnRNP L IP was shown by immunoblot. (C) Protein binding domains of *VEGFA* HSR. Recombinant hnRNP A2/B1, hnRNP L, and DRBP76 were incubated with [^32^P]UTP-labeled *VEGFA* HSR, CARE, AUSL, AUSL-A, and AUSL-D RNA and subjected to UV crosslinking. Crosslinked products were treated with RNase A and detected by SDS-PAGE and autoradiography. (D) HSR region required for RNA switch activity. Reporter constructs containing *FLuc* upstream of wild-type or mutant *VEGFA* HSR were transfected into U937 cells with a plasmid expressing *RLuc* driven by the SV40 promoter. Luciferase activity was measured after treatment with IFN-γ under Nmx. or Hpx. for 8 or 24 h. Relative luciferase activity (FLuc/RLuc) was determined from three independent experiments and reported as mean ± SD (*n* = 3). *FLuc* mRNA expression was determined by semi-quantitative RT-PCR (inset). (E) Permissible spacer length between GAIT element and CARE. Reporter constructs with *FLuc* upstream of wild-type or mutant *VEGFA* HSR containing poly(C) spacers were transfected into U937 cells together with a *RLuc*-bearing plasmid. Luciferase activity was measured after treatment with IFN-γ under Nmx. or Hpx. for 0, 8, and 24 h. Cells were treated as in (D) and luciferase activity determined in three independent experiments, and reported as mean ± SD (*n* = 3). *FLuc* mRNA expression was determined by semiquantitative RT-PCR (inset). (F) Schematic of heterotrimeric HILDA complex binding *VEGFA* HSR RNA in hypoxia.

EPRS and hnRNP L from IFN-γ-treated U937 cells, in either normoxia or hypoxia, bind *in vitro* synthesized *VEGF-A* HSR in a mutually exclusive manner [20]. To provide *in vivo* evidence of the VEGF-A switch, RNA from cells treated with IFN-γ in the presence of normoxia or hypoxia for 24 h were immunoprecipitated with anti-EPRS and -hnRNP L antibodies and subjected to qRT-PCR using transcript-specific primers. GAIT complex EPRS and HILDA complex hnRNP L recognized and bound *VEGFA* mRNA following stimulation by IFN-γ under normoxic and hypoxic conditions, respectively, consistent with previous results (Figure 2A) [20]. To determine whether hnRNP A2/B1 or DRBP76 are required for hnRNP L binding to *VEGFA* mRNA, lysates from cells treated with IFN-γ and hypoxia were subjected to ribonucleoprotein IP (RIP) using anti-hnRNP L antibody, coupled with RT-PCR. hnRNP L interacted with *VEGFA* mRNA in control transfected cells; however, the interaction was substantially reduced following siRNA-mediated depletion of either hnRNP A2/B1 or DRBP76 (Figure 2B). Similarly, the interaction of hnRNP A2/B1 or DRBP76 with *VEGFA* mRNA required the presence of the other (Figure S2, left and center panels). Moreover, the interaction of hnRNP A2/B1 and DRBP76 with *VEGFA* mRNA was abolished following hnRNP L depletion by siRNA-mediated gene silencing (Figure S2, right panels), suggesting that HILDA binding to *VEGFA* mRNA requires integrity of the entire complex. To begin to understand the roles of the individual protein components in RNA switch activity, their binding sites within the HSR region were mapped by UV-crosslinking. Of the three proteins, only hnRNP L and DRBP76 directly bind the *VEGFA* HSR. Interestingly, the two interacting proteins bind different regions of the HSR, hnRNP L binds the CARE, whereas DRBP76 binds the AU-rich stem loop (AUSL) (Figure 2C). The less robust binding to the individual ascending (AUSL-A) and descending (AUSL-D) regions of the AUSL suggests that DRBP76 stabilizes the double-stranded AUSL in a conformation that prevents formation of the GAIT element, which overlaps AUSL-A (Figure 1B). We determined the specific DRBP76-binding region by constructing a series of mutations in either AUSL strand. Mutation of M2 (U^404^ UAUAU ^409^ to AAUAUA), but not M1 (A^416^ AUAUA ^421^ to UUAUAU), inactivated the RNA switch of the HSR-bearing reporter RNA, suggesting the upper stem-loop region of the AUSL is critical (Figure 2D and Figure S3A,B). Differences in luciferase activities of the mutant forms were due largely to altered translation as shown by comparable firefly luciferase mRNA levels determined by semi-quantitative RT-PCR (Figure 2D, insert); renilla luciferase mRNA levels were essentially the same for all transfections (not shown). Complementary covariant mutations (M2–M3, A^381^ UAUAA ^386^ to UAUAUU) on the M3 strand opposing M2 were introduced in an attempt to restore function. However, the M2–M3 double mutant failed to recover RNA switch activity, possibly due to disruption of the GAIT element structure by M2 mutation. Thus, we further created complementary mutations of U^358^ UAUAU ^363^ to AAUAUA (M4) to restore the GAIT element structure at the distal 6-bp stem region. RNA switch activity was partially restored in the M2–M3–M4 triple mutant, indicating the stem structure, not the sequence, is critical for DRBP76 activity in the RNA switch. As controls, individual M3 and M4 mutants lacked GAIT-mediated translational silencing activity and RNA switch function. In the *VEGFA* HSR, the CARE adjoins the GAIT element with not even a single nt separating them (Figure S3A) [20]. To determine the maximum distance between the elements that permits RNA switch activity, we inserted 5- to 25-nt poly(C) spacers between them in an HSR-bearing reporter. Spacers up to 15 nt permitted RNA switch activity, but 20- and 25-nt spacers were inhibitory (Figure 2E and Figure S3A,C), consistent with a distance limit for an effective interaction between the binding proteins hnRNP L and DRBP76. The insertions did not affect mRNA expression of FLuc (Figure 2E, insert) and RLuc (not shown) significantly, indicating that altered translation was responsible for differential Luc activity. Together these results suggest that whereas hnRNP L is responsible for target selectivity, DRBP76, through binding a nearby stem-loop region, has primary responsibility for stabilizing the RNA form lacking the GAIT structural element, thereby suppressing GAIT complex-directed translational silencing (Figure 2F). Knockdown of DRBP76 did not significantly alter *VEGFA* mRNA half-life, providing additional evidence that DRBP76 influences VEGF-A expression primarily at the level of translation (Figure S3D).

**Figure 3:**
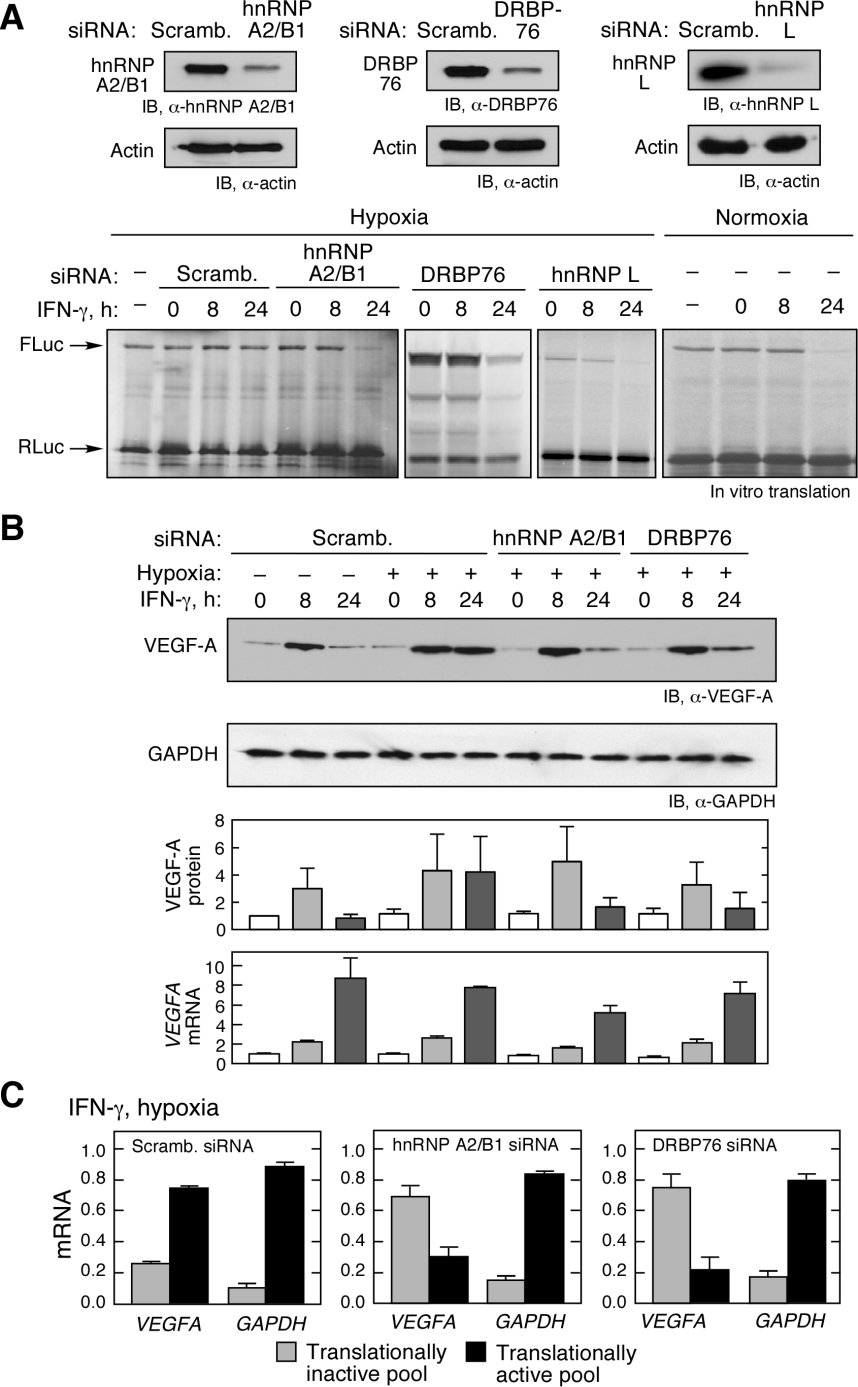
HILDA complex is essential for *VEGFA* RNA switch activity. (A) hnRNP A2/B1, DRBP76, and hnRNP L are required for hypoxia-inducible RNA switch activity *in vitro*. Effectiveness of knockdown by siRNA targeting hnRNP A2/B1, DRBP76, and hnRNP L, and scrambled (scramb.) control was determined by immunoblot analysis; β-actin was probed as loading control (top). *FLuc* reporter RNA bearing the *VEGFA* HSR and *RLuc* control transcripts were subjected to *in vitro* translation in RRL in presence of lysates from U937 cells transfected with scrambled or gene-specific siRNA and incubated with IFN-γ under hypoxia; lysates from IFN-γ-treated normoxic cells shown as control (bottom). (B) hnRNP A2/B1 and DRBP76 are required for robust *in vivo* expression of endogenous VEGF-A in hypoxia. Lysates from siRNA-treated cells as in (A) were probed with anti-VEGF-A and anti-GAPDH antibodies, and normalized VEGF-A expression was quantified by densitometry. Expression of *VEGF-A* mRNA was determined by qRT-PCR and normalized by *GAPDH* mRNA. Results are reported as mean ± SEM (*n* = 3). (C) hnRNP A2/B1 and DRBP76 are required for efficient *VEGFA* mRNA translation in presence of IFN-γ and Hpx. U937 cells were transfected with siRNA targeting hnRNP A2/B1 and DRBP76 (or scrambled siRNA), and then subjected to IFN-γ and Hpx. Cell lysates were fractionated on a sucrose gradient, and total RNA in translationally active and inactive pools subjected to qRT-PCR with *VEGFA*- and *GAPDH*-specific primers. Results are reported as mean ± SD (*n* = 3).

By knockdown and overexpression experiments, we previously reported that hnRNP L is essential for hypoxia-induced switch activity in U937 cells [20]. To test the requirement for the other HILDA components, DRBP76 and hnRNP A2/B1, both were subjected to siRNA-mediated knock-down (hnRNP L knock-down served as positive control) (Figure 3A, top). Cells were treated with IFN-γ and hypoxia for up to 24 h, and lysates tested for their effect on *in vitro* translation of an HSR-bearing reporter. As seen before, 24-h lysates from IFN-γ-treated normoxic cells inhibited translation of the reporter, but 24-h lysates from hypoxic cells were inactive (Figure 3A, bottom). However, deletion of either DRBP76 or hnRNP A2/B1 dramatically impaired the hypoxia-driven RNA switch to an extent comparable to that of hnRNP L knockdown, and permitted GAIT complex-mediated translation inhibition by 24-h lysates (Figure 3A, bottom). We investigated the effect of these lysates on endogenous gene expression. As before, hypoxia prevented IFN-γ-mediated inhibition of expression of VEGF-A observed at 24 h (Figure 3B). However, siRNA-mediated knock-down of either DRBP76 or hnRNP A2/B1 restored translational inhibition of VEGF-A without significantly altering the steady-state level of *VEGFA* mRNA (Figure 3B). Polysome profiling was done to verify that the effects on VEGF-A expression were due to altered translation. IFN-γ activation of the GAIT pathway inhibited *VEGF-A* mRNA translation-initiation [21], and this inhibition was reversed by hypoxia [20]. Indeed, following IFN-γ treatment under hypoxia, knock-down of either hnRNP A2/B1 or DRBP76 induced a dramatic shift of endogenous *VEGFA* mRNA from translationally active polysome pools to translationally inactive free mRNP pools (Figure 3C and Figure S4).

### IFN-γ Induces von Hippel-Lindau-mediated Degradation of hnRNP L

**Figure 4:**
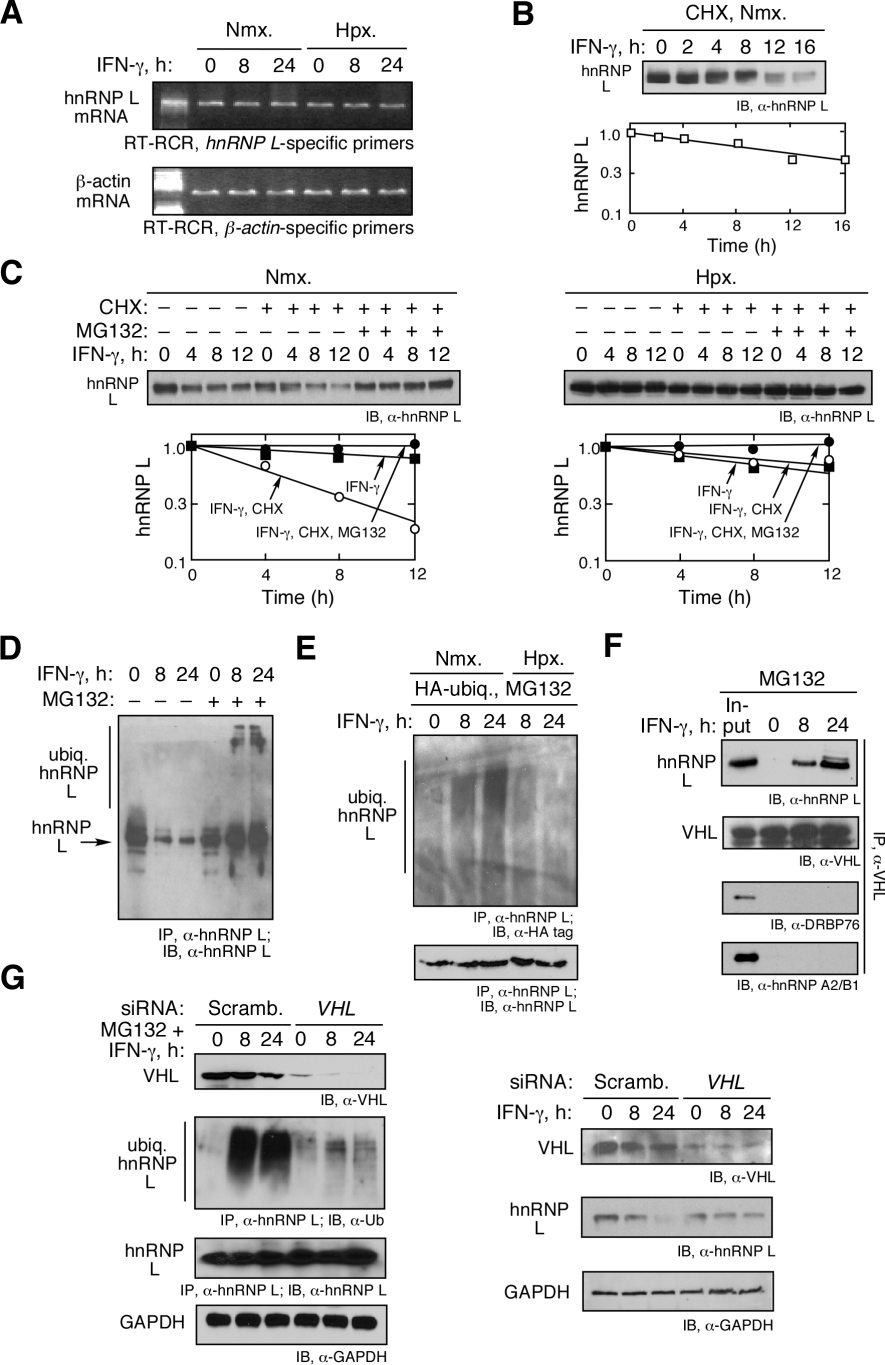
IFN-γ induces VHL-mediated polyubiquitination and degradation of prolylhydroxylated hnRNP L. (A) Steady-state amount of hnRNP L mRNA is not regulated by IFN-γ. U937 cells were treated with IFN-γ under Nmx. or Hpx. for 0, 8, and 24 h. *HnRNP L* and *β-actin* mRNA were determined by semiquantitative RT-PCR. (B) IFN-γ induces hnRNP L degradation in normoxic cells. U937 cells were treated with CHX for up to 16 h under Nmx. and lysates subjected to immunoblot and quantitated by densitometry. (C) IFN-γ-inducible degradation of hnRNP L is proteasome-mediated. U937 cells were treated with CHX or CHX plus MG132 in presence of IFN-γ for up to 12 h under Nmx. (left panel) or Hpx. (right panel), and lysates subjected to immunoblot and quantitated by densitometry. (D) IFN-γ induces polyubiquitination of endogenous hnRNP L *in vivo*. U937 cells were treated with IFN-γ for up to 24 h in the absence or presence of MG132, and lysates subjected to IP with mouse-derived hnRNP L antibody followed by immunoblot with rabbit-derived hnRNP L antibody. (E) IFN-γ induces normoxia-dependent ubiquitination of hnRNP L. U937 cells were transfected with HA-ubiquitin, treated with MG132 in Nmx. or Hpx., immunoprecipitated with anti-hnRNP L antibody, and subjected to immunoblot with anti-HA antibody. (F) IFN-γ induces interaction of hnRNP L with VHL. Lysates from U937 cells treated with IFN-γ and MG132 for up to 24 h were immunoprecipitated with anti-VHL antibody and subjected to immunoblot with anti-hnRNP L, -VHL, -hnRNP A2/B1, and -DRBP76 antibodies. (G) IFN-γ-induced polyubiquitination and degradation of hnRNP L is mediated by VHL. U937 cells were transfected with VHL-specific (or scrambled) siRNA. After recovery, cells were treated with IFN-γ in the presence or absence of MG132 and lysates immunoblotted with anti-VHL, -hnRNP L, -ubiquitin, and -GAPDH antibodies.

hnRNP L expression is markedly reduced in normoxic, IFN-γ-treated cells, thereby permitting GAIT complex binding to the *VEGFA* mRNA and transcript-specific translational silencing [20]. Semiquantitative RT-PCR (Figure 4A) and Northern blot analysis (Figure S5) showed that hnRNP L mRNA expression is unaltered by either hypoxia or IFN-γ treatment for up to 24 h, and that altered hnRNP L expression must be posttranscriptional. hnRNP L half-life was measured in the presence of cycloheximide to inhibit protein synthesis. In nonstressed monocytic cells (normoxia, no IFN-γ) the half-life of hnRNP L is about 12 h (Figure 4B and Figure S6A). The half-life of hnRNP L was shortened to about 4 h by IFN-γ treatment in normoxia; however, hypoxia suppressed the effect of IFN-γ, restoring the half-life to about 12 h (Figure 4C and Figure S6B). As shown previously, the proteasome inhibitor MG132 blocked IFN-γ-mediated hnRNP L degradation, indicating an important role of the ubiquitin/proteasome pathway in regulating hnRNP L expression [20].

To investigate the mechanism underlying IFN-γ-induced hnRNP L degradation, hnRNP L ubiquitination was determined. IFN-γ treatment in the presence of MG132 induced accumulation of a high molecular weight form of hnRNP L consistent with ubiquitination (Figure 4D). Expression of HA-ubiquitin and detection with anti-HA-tag antibody confirmed formation of high molecular weight, ubiquitinated hnRNP L, and exposure to hypoxia dramatically diminished hnRNP L ubiquitination (Figure 4E). We considered the von Hippel-Lindau (VHL)-containing ubiquitin ligase complex as a candidate E3 ubiquitin-protein ligase because of its normoxia-dependent role in regulation. VHL specifically targets proteins, e.g., hypoxia inducible factor (HIF)-1α tagged by O_2_-dependent prolyl hydroxylation [22]. VHL was shown to interact robustly with hnRNP L, but not with hnRNP A2/B1 or DRBP76, in an IFN-γ-dependent manner (Figure 4F). Also, siRNA-mediated knockdown of VHL markedly reduced hnRNP L polyubiquitination (Figure 4G, left panel) with MG132 treatment, and increased hnRNP L stability following IFN-γ treatment in absence of MG132 (Figure 4G, right panel). However, overexpression of VHL did not affect the stability of hnRNP L or the assembly of the HILDA complex in hypoxia, suggesting that HILDA complex formation might contribute to protection of hnRNP L from VHL-mediated degradation (Figure S7). In an *in vitro* ubiquitination system reconstituted with exogenous E1 and E2 enzymes and E3 ubiquitin ligase pVHL derived from lysate of 8 h, IFN-γ-treated U937 cells in normoxia further confirmed robust polyubiquitination of hnRNP L (Figure S8). In contrast, cell lysate from hypoxia-treated U937 cells failed to modify hnRNP L. Similar results were obtained with primary human PBM (not shown). These results suggest that proteasomal degradation of hnRNP L in U937 cells and in human PBM is mediated by IFN-γ-triggered ubiquitination by a VHL-containing E3 ubiquitin ligase.

### Hypoxia-Induced Phosphorylation of hnRNP L at Tyr^359^ Promotes Cytoplasmic Localization and Inhibits Degradation

**Figure 5:**
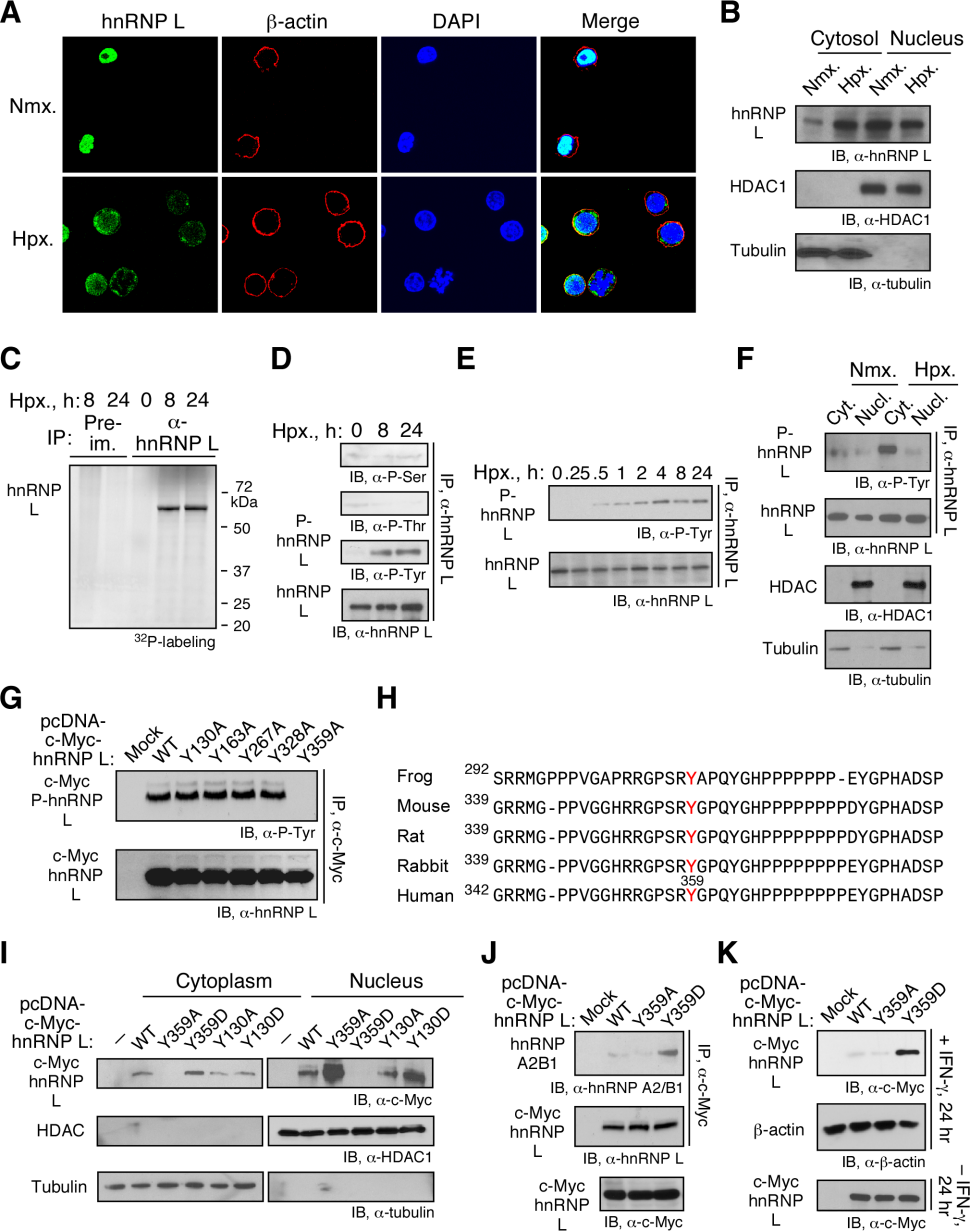
Hypoxia-inducible hnRNP L phosphorylation at Tyr^359^ suppresses nuclear translocation and cytoplasmic degradation. (A) Hypoxia increases cytoplasmic localization of hnRNP L. U937 cells treated with IFN-γ for 24 h under Nmx. or Hpx. were immunostained using rabbit anti-hnRNP L and -β-actin antibodies. Cell nuclei were stained with DAPI. (B) Analysis of hypoxia-stimulated translocation of hnRNP L by cell fractionation. U937 cells treated with IFN-γ in Nmx. or Hpx. were fractionated and subjected to immunoblot. (C) Hypoxia induces hnRNP L phosphorylation *in vivo*. U937 cells were incubated under Hpx. and then with a 4-h pulse of ^32^P-orthophosphate between 6 and 10 h (denoted as 8 h) or between 22 and 26 h (denoted as 24 h). Lysates were immunoprecipitated with anti-hnRNP L antibody (or pre-immune IgG), and ^32^P-labeled protein detected by autoradiography. (D) Hypoxia induces tyrosine phosphorylation of hnRNP L. Lysates from cells treated with Hpx. for 0, 8, and 24 h were immunoprecipitated with anti-hnRNP L antibody and immunoblotted with antibodies targeting phosphoserine (P-Ser), phosphothreonine (P-Thr), or phosphotyrosine (P-Tyr). (E) Time course of hypoxia-inducible hnRNP L phosphorylation. U937 cells were treated with Hpx. for up to 24 h and lysates immunoprecipitated with anti-hnRNP L antibody and immunoblotted with antibodies targeting P-Tyr or hnRNP L. (F) Hypoxia induces cytoplasmic accumulation of P-Tyr-hnRNP L. Cytosolic and nuclear fractions from U937 cells treated with Hpx. for 24 h were immunprecipitated with anti-hnRNP L antibody and immunoblotted with anti-P-Tyr and -hnRNP L antibodies. Western blots were done using anti-HDAC1 and anti-tubulin antibodies. (G) Hypoxia induces Tyr^359^ phosphorylation of hnRNP L. pcDNA3-hnRNP L-Myc bearing selected Tyr-to-Ala mutations were transfected into U937 cells with endogenous hnRNP L knocked down by 3′UTR-targeting siRNA. After recovery, cells were treated with Hpx. for 24 h. Lysates were immunprecipitated with anti-hnRNP L antibody and immunoblotted with anti-P-Tyr and -hnRNP L antibodies. (H) Sequence conservation of hnRNP L phospho-site in vertebrate animals. Tyr phospho-site in aligned sequences is shown (red). (I) Cellular localization of phospho-mimetic and phospho-dead hnRNP L in normoxia. c-Myc-tagged, wild-type (WT), phospho-mimetic (Tyr-to-Asp, Y-D), and phospho-dead (Tyr-to-Ala, Y-A) mutant hnRNP L were transiently transfected into U937 cells and were determined in cytoplasmic and nuclear fractions by immunoblot analysis with anti-c-Myc, -HDAC1, and -tubulin antibodies. (J) Phospho-mimetic hnRNP L binds hnRNP A2/B1. c-Myc-tagged, wild-type (WT), phospho-dead (Y-A), and phospho-mimetic (Y-D) hnRNP L were expressed in U937 cells by transient transfection. Lysates were immunoprecipitated with anti-c-Myc antibody and immunoblotted with anti-hnRNP L and -hnRNP A2/B1 antibodies. Expression of c-Myc-tagged hnRNP L was determined with anti-c-Myc antibody of total lysate. (K) Phospho-mimetic hnRNP L inhibits VHL-mediated, proteasomal degradation of hnRNP L. c-Myc-tagged wild-type, phospho-dead, and phospho-mimetic hnRNP L was transiently transfected into U937 cells, and then treated with IFN-γ for 24 h. Lysates were immunoblotted with anti-c-Myc and -actin antibodies. C-Myc-tagged hnRNP L was determined by immunoblot with anti-c-Myc antibody of total lysate from cells not treated with IFN-γ as controls for transfection and expression of hnRNP L-bearing vectors.

hnRNP L is primarily localized in the nucleus in human monocytic cells but substantially redistributes to the cytoplasm during hypoxia [23]. Fluorescence visualization verified hypoxia-driven cytoplasmic relocalization of hnRNP L, even in the presence of IFN-γ (Figure 5A). Similar hypoxia-stimulated cytoplasmic relocalization of hnRNP L was observed in primary human PBM-derived macrophages induced by macrophage colony stimulating factor (M-CSF) (Figure S9). Immunoblot analysis of cytosolic and nuclear fractions from IFN-γ- and hypoxia-treated cells further confirmed hnRNP L translocation (Figure 5B). Cellular localization of RBPs can be regulated by their phosphorylation state [24]–[26]. Metabolic labeling with ^32^P-orthophosphate showed that hypoxia induced robust phosphorylation of hnRNP L at 8 h, and the modification was stable for at least 24 h (Figure 5C). Immunoblot analysis of hnRNP L immunoprecipitated from hypoxia-treated cells with phospho-specific antibodies revealed strong phosphorylation at Tyr, but not at Ser or Thr (Figure 5D). A time course experiment showed modest hnRNP L Tyr-phosphorylation after 0.5 h of hypoxia and maximal phosphorylation after 4 h in U937 cells (Figure 5E) and in primary human PBM (not shown). Immunoblot analysis with anti-pTyr antibody showed Tyr-phosphorylated hnRNP L was almost completely restricted to the cytoplasm in hypoxia-treated cells (Figure 5F).

To identify the hypoxia-induced phosphorylation site, hnRNP L was immunoprecipitated from lysates of hypoxia-treated cells, and phospho-sites detected by mass spectrometry. Total coverage with three protease treatments was 84%, but phosphorylation events were not detected (Figure S10). Endogenous hnRNP L in U937 cells was knocked down with siRNA targeting the 3′UTR, and cells transfected with cDNA constructs containing specific, site-directed Tyr-to-Ala mutations at residues in regions not covered by the mass spectrometry analysis. Among the five hnRNP L mutants tested, only Y359A was not phosphorylated in U937 monocytic cells (Figure 5G) and in human PBM (not shown). Tyr^359^, and the surrounding sequence, is evolutionarily conserved from frogs to humans (Figure 5H), and has been identified as a phospho-site by high-throughput proteomic survey (www.phosphosite.org) in both mouse and human (in addition to Tyr phosphorylation at positions 47, 48, 92, 267, 285, 333, 340, 363, 375, 565, 574, and 576). To determine the role of Tyr^359^ phosphorylation in hnRNP L localization, cells were transfected with c-Myc-tagged wild-type cDNA or, phospho-dead (Y359A) or phospho-mimetic (Y359D) mutants. Under normoxic conditions, wild-type hnRNP L is primarily localized in the nucleus, but also present in the cytoplasm, as observed previously [23]. In contrast, the Y359A mutant was exclusively in the nucleus, and the Y359D mutant was exclusively cytoplasmic (Figure 5I). Similarly, following IFN-γ stimulation under hypoxia, the Y359A and Y359F hnRNP L mutants were exclusively localized in the nucleus (Figure S11). As a control for specificity, Tyr^130^ mutants did not partition with the Tyr^359^ mutants. Cells were transfected with c-Myc-tagged wild-type or mutant hnRNP L, immunoprecipitated with anti-c-Myc antibody, and probed with hnRNP A2/B1 antibody. Y359D exhibited much greater binding to hnRNP A2/B1 compared to wild-type or Y359A mutant hnRNP L (Figure 5J). Remarkably, the Y359D mutant, but not the Y359A mutant or wild-type protein, was completely resistant to IFN-γ-stimulated degradation (Figure 5K). Consistent with the cellular translocation of hnRNP L (Figure 5A), Tyr phosphorylation was induced by IFN-γ treatment in hypoxia (Figure S12). In summary, hypoxia-inducible Tyr^359^ phosphorylation of hnRNP L facilitates its cytoplasmic relocalization and prevents its degradation.

### HnRNP A2/B1 Binds and Protects hnRNP L from IFN-γ-Induced Prolyl Hydroxylation and VHL-Mediated Degradation

**Figure 6:**
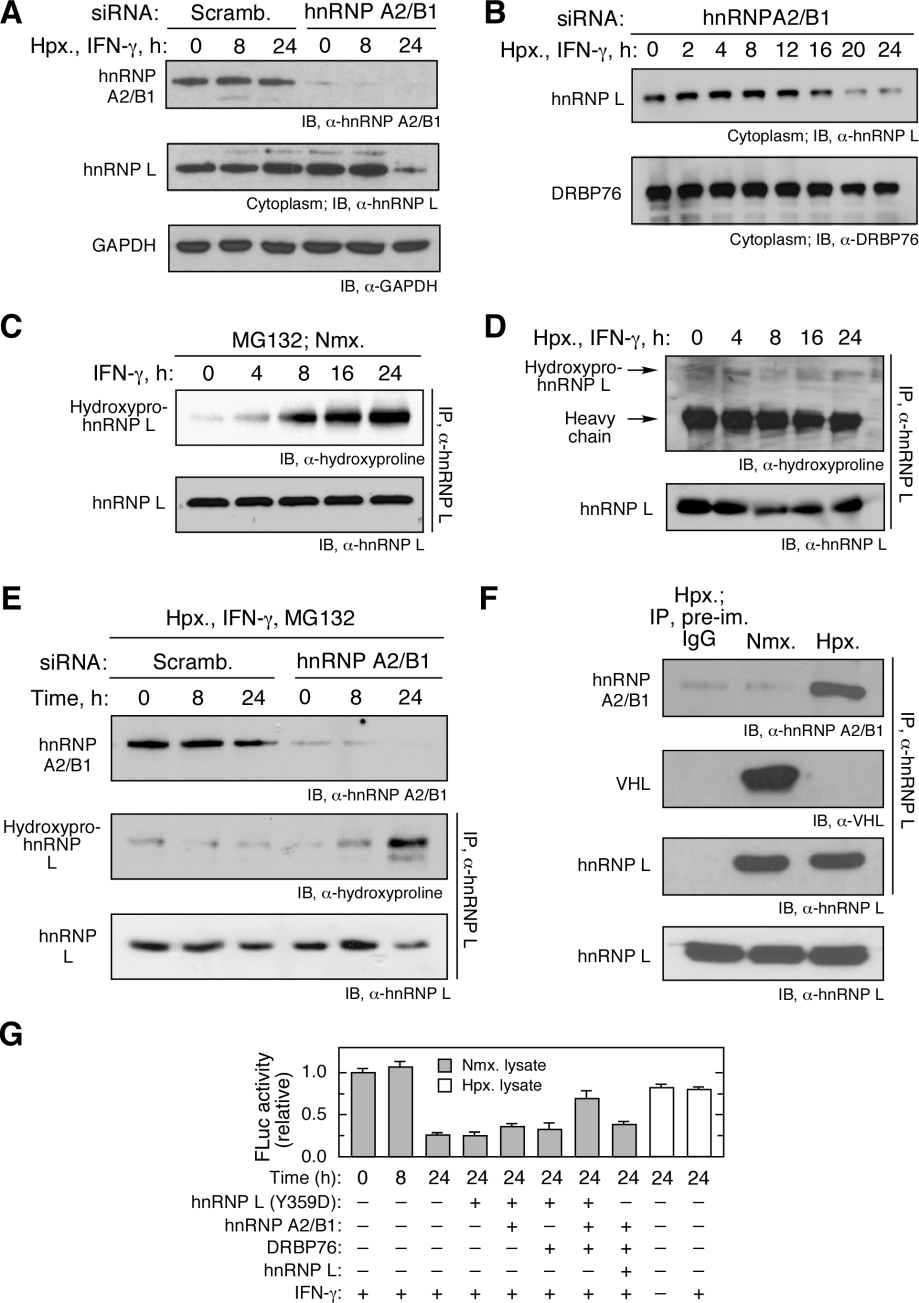
hnRNP A2/B1 prevents IFN-γ-induced hnRNP L prolyl hydroxylation, blocks interaction with VHL, and stabilizes hnRNP L. (A) Rapid degradation of hnRNP L in absence of hnRNP A2/B1. U937 cells were transfected with hnRNP A2/B1-specific (or scrambled) siRNA. After recovery, cells were treated with IFN-γ and Hpx. for 0, 8, and 24 h. Lysates were immunoblotted with anti-hnRNP A2/B1, -hnRNP L, and -GAPDH antibodies. (B) Time course of hnRNP L degradation in absence of hnRNP A2/B1. U937 cells were treated as in (A) for up to 24 h. Lysates were immunoblotted with anti-hnRNP L and -DRBP76 antibodies. (C) IFN-γ induces prolyl hydroxylation of hnRNP L. U937 cells were treated with IFN-γ and MG132 for up to 24 h. Lysates were immunoprecipitated with anti-hnRNP L antibody and immunoblotted with anti-hydroxyproline and -hnRNP L antibodies. (D) hnRNP L is not subject to prolyl hydroxylation in Hpx. U937 cells were treated with IFN-γ and Hpx. for up to 24 h. Lysates were immunoprecipitated with anti-hnRNP L antibody and immunoblotted with anti-hydroxyproline and -hnRNP L antibodies. (E) hnRNP A2/B1 inhibits prolyl hydroxylation of hnRNP L in hypoxia. U937 cells were transfected with hnRNP A2/B1-specific (or scrambled) siRNA. After recovery, cells were treated with IFN-γ, Hpx., and MG132 for up to 24 h. Lysates were immunoblotted with anti-hnRNP A2/B1 antibody. Lysates were also immunoprecipitated with anti-hnRNP L antibody and immunoblotted with anti-hydroxyproline and -hnRNP L antibodies. (F) Hypoxia induces binding of hnRNP A2/B1 to hnRNP L and prevents VHL binding. U937 cells were subjected to Nmx. or Hpx. for 24 h in the presence of IFN-γ stimulus. Lysates were immunoprecipitated with anti-hnRNP L antibody and immunoblotted with anti-hnRNP A2/B1 and -VHL antibodies. Total hnRNP L in cell lysates was determined by immunoblot. IP with pre-immune IgG of hypoxic lysate served as a control. (G) Reconstitution of RNA switch function of HILDA complex. Phospho-mimetic hnRNP L (Y359D) was pre-incubated with DRBP76 and hnRNP A2/B1 as indicated (5 pmol each) for 0.5 h on ice. *In vitro* translation of the FLuc reporter bearing the *VEGFA* HSR element (and *RLuc* control RNA) was determined in a wheat germ extract in the presence of ^35^S-Met, cytosolic extracts from IFN-γ-treated U937 cells, and HILDA components as indicated. In a control experiment, wild-type hnRNP L replaced phospho-mimetic hnRNP L. FLuc expression was normalized by RLuc and reported as mean ± SD, *n* = 3.

Because hnRNP A2/B1 does not bind the HSR directly, it is more likely involved in regulation of its binding partner hnRNP L, than in operating the RNA switch itself. We tested the possibility that hnRNP A2/B1 contributes to hypoxia-induced stabilization of hnRNP L. siRNA-mediated knockdown of hnRNP A2/B1 resulted in hnRNP L destabilization following IFN-γ treatment in hypoxia (Figure 6A). In contrast, hnRNP A2/B1 knockdown did not induce DRBP76 degradation (Figure 6B). Also, siRNA-mediated knockdown of DRBP76 did not affect hnRNP L stability (Figure S13). Interestingly, hnRNP L was subject to IFN-γ-dependent Pro hydroxylation as shown by IP followed by probing with anti-hydroxyproline antibody (Figure 6C). Hypoxia prevented the IFN-γ-inducible prolyl hydroxylation of hnRNP L (Figure 6D). Knockdown of hnRNP A2/B1 under hypoxic condition and in the presence of IFN-γ and MG132 restored marked Pro hydroxylation of hnRNP L after 24 h (Figure 6E). Finally, co-IP with anti-hnRNP L antibody revealed that hypoxia induced hnRNP A2/B1 binding to hnRNP L, and completely blocked hnRNP L recognition by VHL (Figure 6F). These results indicate that the major function of hnRNP A2/B1 in the heterotrimeric switch is to protect hnRNP L from IFN-γ-triggered prolyl hydroxylation, ubiquitination, and subsequent degradation. Treatment of U937 cells with prolyl hydroxylase (PH) inhibitors L-mimosine and dimethyloxalylglycine (DMOG) blocked prolyl hydroxylation of hnRNP L and caused marked stabilization of the protein in the presence of IFN-γ under normoxia (Figure S14).

### *In Vitro* Reconstitution of HILDA Complex and RNA Switch Activity

Co-IP and RNA-binding studies suggest a model in which the interaction between DRBP76 and hnRNP A2/B1 is indirect and facilitated by hnRNP L and *VEGFA* HSR RNA (Figure 2F). We investigated the interactions in detail by *in vitro* reconstitution using recombinant proteins and *in vitro*–transcribed RNA. DRBP76 and hnRNP A2/B1 by themselves did not bind, nor did the addition of either hnRNP L or HSR RNA restore their interaction significantly (Figure S15). However, when both hnRNP L and HSR RNA were added, then a modest interaction between hnRNP A2/B1 and DRBP76 was detected. A much stronger interaction was observed when phospho-mimetic hnRNP L (Y359D) was added together with HSR RNA, but not nonspecific RNA, thereby reconstituting the entire HILDA complex *in vitro*. To investigate the sufficiency of hnRNP L, hnRNP A2/B1, and DRBP76 in operating the RNA switch, we determined the regulatory activity of the purified proteins *in vitro*. Phospho-mimetic hnRNP L (Y359D) was used to facilitate interaction with hnRNP A2/B1. The three proteins were pre-incubated in several combinations, and their effect on *in vitro* translation of an FLuc reporter bearing the *VEGFA* HSR element (and RLuc control RNA) was determined in a wheat germ extract in the presence of ^35^S-Met and cytosolic extracts from IFN-γ-treated U937 cells. hnRNP L (Y359D) by itself or with either hnRNP A2/B1 or DRBP76, did not restore translation in the presence of lysates from cells treated with IFN-γ for 24 h (Figure 6G). However, the three proteins together substantially overcame the translational inhibition. Substitution of wild-type hnRNP L for the phospho-mimetic was ineffective, suggesting the posttranslational modification is not only required for maintaining a high level of cytoplasmic hnRNP L, but also is required for HILDA complex assembly. As positive controls, lysates from cells treated for 24 h with or without IFN-γ in hypoxia could rescue translation of HSR-bearing FLuc. These results support the role of the heterotrimeric HILDA complex in operating the RNA conformational switch.

## Discussion

### HILDA Complex Directs the *VEGFA* mRNA Switch

The combinatorial activity of pairs of nearby elements has become an area of increasing interest, particularly with the recent recognition that microRNA binding to targets can influence protein binding to nearby target RNA elements [27]. There are few cases in which pairs of protein-binding RNA elements dictate the response. In one well-studied example, a combinatorial code in which the number and position of two elements—namely, the cytoplasmic polyadenylation element and Pumilio-binding element—determine translational activation or repression in *Xenopus* oocytes [28]. However, there is a dearth of studies on the mechanisms by which nearby RNA elements, and their cognate binding factors, integrate disparate environmental signals to generate a binary response and regulate gene expression. In one known case, the leader sequence of the Mg^2+^ transporter gene mgtA of *Salmonella enterica* contains a Mg^2+^-sensing riboswitch and an 18-codon, proline- or hyperosmotic stress-sensing ORF that integrate distinct signals to generate the cell response; however, an interaction between the disparate elements was not observed [29]. In the case of the GAIT system, we have reported that hypoxia prevents GAIT complex binding to the *VEGFA* 3′UTR by a switch in the conformation of RNA that masks the GAIT structural element [20] by converting the element into the ascending half of a long, double-stranded stem-loop. The switch is initiated by hypoxia-stimulated binding of hnRNP L to a 3′UTR CARE directly adjacent to the GAIT element. In this report we define the components of a heterotrimeric complex that constitutes the RNA switch, their regulation by IFN-γ and hypoxia, and their specific functions in directing the *VEGFA* mRNA switch in human monocytic cells.

The requirement for each of the components of the HILDA complex to drive the RNA switch was shown by knockdown experiments in cells, and their sufficiency shown by in vitro reconstitution. The HILDA complex has not been previously described, but its individual components are known to regulate distinct mRNA-related functions. DRBP76 was initially identified through its binding to double-stranded RNA and to protein kinase R (PKR) [30]. DRBP76 exhibits multiple RNA-related functions including regulation of transcription, mRNA stability [31], and translation [32]. DRBP76 also binds the *VEGFA* HSR in hypoxic breast cancer cells, increasing mRNA stability and translation, but the binding region within the *VEGFA* HSR in these experiments was not determined [33]. The double-stranded RNA-binding property of DRBP76 is most likely the critical function it performs in the context of the HILDA complex, stabilizing the conformation featuring a long, double-stranded stem loop, and disrupting the structure of the GAIT element. hnRNP A2/B1, like hnRNP L, participates in splicing of pre-mRNAs and in translational regulation [34]. hnRNP A2/B1 also serves as a molecular motor-powered transporter of select mRNAs bearing specific hnRNP A2/B1 response elements (A2RE), for example, neurogranin, Arc, and calmodulin-dependent kinase II [35]–[37]. Cytosolic complexes containing heterodimeric hnRNPs have been shown to interact with specific target mRNAs. For example, hnRNP L and I form a complex that binds murine inducible nitric oxide synthase mRNA, and regulates its translation [38]. Interestingly, the same pair of hnRNPs found in the HILDA complex, hnRNP L and A2/B1, interacts with the glucose transporter 1 (Glut1) 3′UTR, inducing translational repression and mRNA instability [18]. However, an interaction between DRBP76 and A2/B1 has not been described.

### hnRNP L Is Subject to Dual Regulation by IFN-γ and Hypoxia

**Figure 7:**
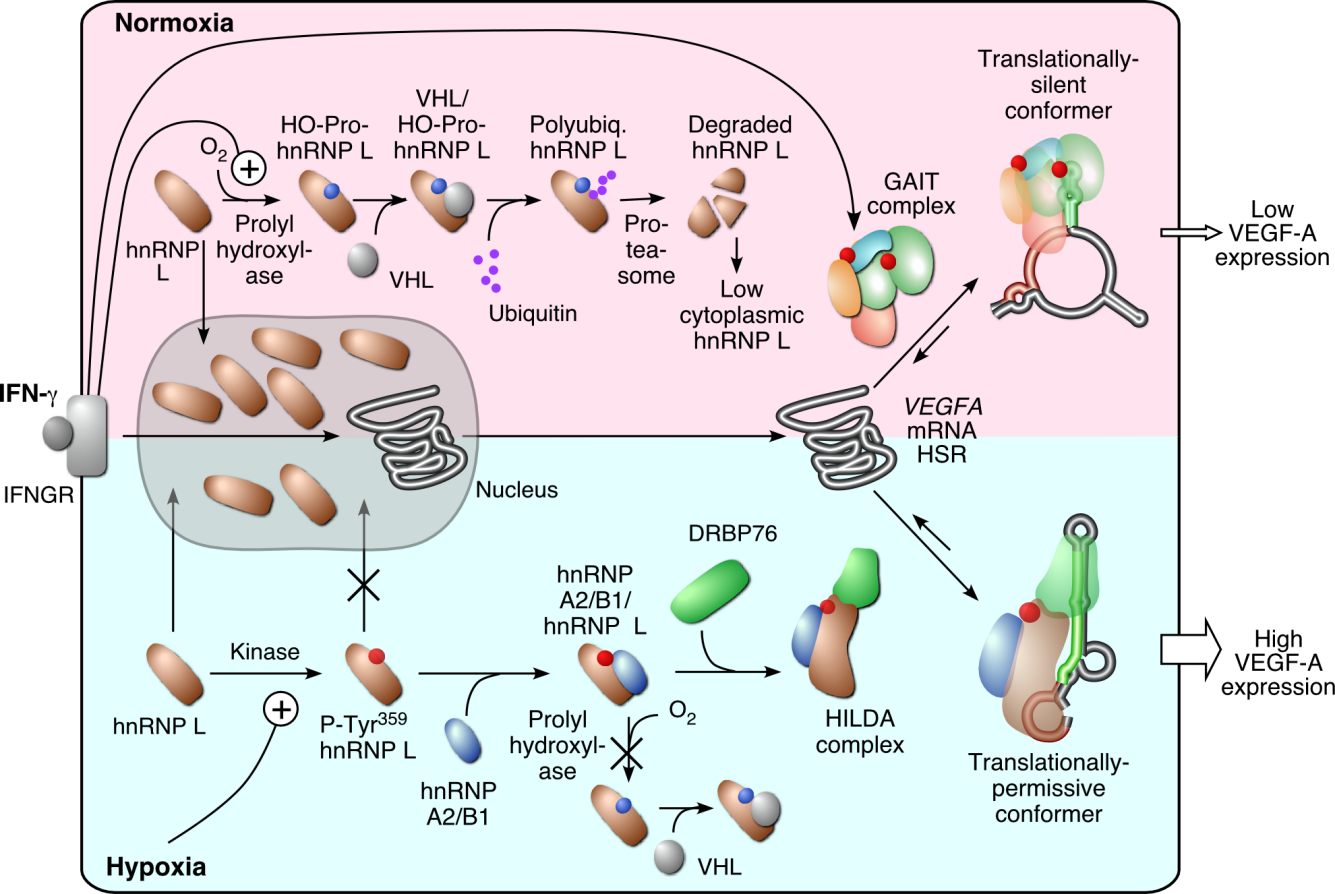
Regulation of hnRNP L expression by IFN-γ and hypoxia and the role of the HILDA complex in the *VEGFA* RNA switch.

hnRNP L is a critical component of the HILDA complex because it is uniquely responsible for stimulus sensing as well as target recognition. Our results show that the steady-state level and cellular localization of hnRNP L in myeloid cells are regulated both by IFN-γ and by hypoxia. Under normoxic conditions hnRNP L is distributed between the cytoplasm and nucleus, the latter for execution of mRNA processing functions. IFN-γ induces prolyl hydroxylation of cytoplasmic hnRNP L and consequent rapid, VHL-mediated ubiquitination and proteasomal degradation (Figure 7). Near-complete cytoplasmic depletion of hnRNP L permits GAIT complex binding to the *VEGFA* GAIT element in the translationally silent conformer, resulting in low-level translation of *VEGFA* mRNA. Hypoxia induces phosphorylation of hnRNP L on Tyr^359^, which increases cytoplasmic localization by restricting transport into the nucleus. Hypoxia-inducible phosphorylation suggests the activity of a nonreceptor Tyr kinase such as a member of the Src, Abl, Jak, Syk, or Fak families. The sequence surrounding the Tyr^359^ phosphorylation site (pRRGPSR^359^ **Y**GPQYGHPPPPPPPP) exhibits 100% conservation in humans, rodents, rabbits, and frogs, and provides insight into the identity of the proximal kinase. “YG” is a specific Src kinase substrate motif (PhosphoMotif Finder), and the downstream polyproline motif is a binding site for SH3-containing proteins, including Src family kinases. hnRNP A2/B1 binds Tyr^359^-phosphorylated hnRNP L and blocks recognition by VHL-containing E3 ubiquitin ligase complex, thus permitting cytoplasmic accumulation. The precise kinetics and binding order have not been determined, but our results suggest that the phospho-hnRNP L and hnRNP A2/B1 recruit DRBP76 to form the heterotrimeric HILDA complex that binds the *VEGFA* CARE. The interaction is weakened by nuclease treatment, indicating that the binding of DRBP76 to other complex members is enhanced by its interaction with the long, AU-rich stem-loop within the *VEGFA* HSR. The HILDA complex stabilizes the translationally permissive conformer that masks the GAIT element, thus resulting in uninhibited translation of *VEGFA* mRNA, even in the presence of IFN-γ-induced GAIT complex.

The tumor suppressor protein VHL is an essential, target-specific component of a multifunctional E3 ubiquitin ligase complex involved in protein degradation [39]. The best-known target of VHL is hypoxia inducible factor (HIF)-1α and -2α, transcription factors that stimulate expression of multiple hypoxia-inducible transcripts, including *VEGFA* mRNA. In normoxia, O_2_-dependent prolyl hydroxylation of HIF-1α triggers recognition by VHL and consequent degradation, thereby inhibiting expression of HIF-1α targets [40]. However, prolyl hydroxylation of HIF-1α is inhibited in hypoxia, thereby stabilizing HIF-1α and increasing target mRNA transcription. Other VHL targets have been identified in renal cell carcinoma cell lines; interestingly, several are downregulated by VHL [41]–[43]. hnRNP A2/B1 has been reported to be targeted by VHL [44]. However, we find hnRNP A2/B1 binding to hnRNP L prevents targeting by VHL in human monocytic cells. Possibly, cell-type specificity of targets and directionality of regulation—i.e., up or down—are promoted by additional factors within the VHL-bearing E3-ubiquitin ligase complex. Proline hydroxylase inhibitors DMOG and L-mimosine both block hnRNP L prolyl hydroxylation and consequent degradation. Collagen prolyl-4-hydroxylase (C-P4H) is a candidate because it is induced by hypoxia [45],[46] and hydroxylates and destabilizes another RBP, Argonaute 2 (Ago2) [47]. Likewise, HIF prolyl hydroxylase (HIF-PH) is a candidate because it modifies HIF-1α for poly-ubiquitination by pVHL and proteasomal degradation [48].

### HILDA Complex as Archetype Protein-Directed RNA Switch Flipper in Vertebrates

Long, noncoding regions of mRNAs, because of their manifold protein- and RNA-binding elements, are potentially ideal for integration of multiple inputs into a single output—i.e., gene expression. Because of their unusually long length, the 3′UTR, which averages almost 600 nt in human mRNAs versus about 150 nt for 5′UTRs, is a particularly attractive target for signal integration [49]. A plethora of examples of posttranscriptional regulation have been described in which RBPs are activated by environmental signals that alter their binding behavior, generally by posttranslational modification and complex formation [50]. In most known cases, RBPs or complexes interact one-to-one with preformed sequence or structural elements [50],[51]. More recently, regulatory processes have been described in which signals alter the conformation of the RNA to modulate gene expression [52]. The *VEGFA* 3′UTR RNA switch features alternative interaction of distinct protein complexes in response to environmental signals, culminating in regulated gene expression. The CARE element is analagous to a riboswitch aptamer domain, and hnRNP L acts as a “responder/selector,” responding to environmental cues and determining HILDA complex mRNA target specificity. The AUSL element determines the expression outcome: VEGF-A expression is high when the double-stranded conformation is bound by the HILDA complex, and expression is depressed when the GAIT complex binds the GAIT element in the alternate conformation (Figure 7). To our knowledge there are not any previous reports of 3-RNA element switches. Likewise, the integration of two different signals—i.e., hypoxia and inflammatory cytokine—by the *VEGFA* RNA switch lacks precedent.

The principles, protein constituents, and mechanisms utilized by the *VEGFA* switch might be applicable to distinct mRNA switches. One possibility is that the HILDA complex recognizes other transcripts with sequence and structural elements analogous to the *VEGFA* switch region—i.e., CARE and GAIT elements nearby DRBP76-binding double-stranded RNA stretches. Cytoplasmic hnRNP L binds VEGF-A mRNA and other transcripts in multiple cell lines [18],[19],[38], suggesting that the HILDA complex might direct additional RNA switches. More generally, distinct RBPs may replace hnRNP L as the “specificity factor,” but likewise recruit DRBP76 to stabilize nearby stem-loop structures and drive formation of alternate regulatory conformers. High-throughput screening has identified at least two RBPs hnRNP A1 and FUS (fused in sarcoma) that bind DRBP76 and might direct alternate RNA switches [53],[54]. Alternatively, other inhibitory factors (microRNA or proteins) might replace the GAIT complex to drive the hnRNP L-directed GAIT-independent RNA switches in more general sense. We speculate that *the VEGFA* switch is a founding member of signal-activated, protein-directed, RNA switches that regulate posttranscriptional gene expression in vertebrates, and similar switches might be widespread RNA sensors in multicellular animals.

## Materials and Methods

### Reagents

Phospho-safe extraction buffer was from Novagen (Madison, WI). Rabbit reticulocyte lysate, wheat germ extract, large-scale RNA production system-T7, and dual luciferase reporter assay system were from Promega (Madison, WI). Human IFN-γ was obtained from R&D Systems (Minneapolis, MN). Human monocyte nucleofactor kit was from Lonza (Switzerland). Reagents for protein purification, nuclear and cytosolic extraction, and immunoanalysis were from Pierce (Rockford, IL). Primers, dNTP mix, TRIzol LS reagent, one-step RT-PCR system, and competent cells were from Invitrogen (Carlsbad, CA). Protein A/G beads, anti-α-tubulin, anti-hnRNP A2/B1, rabbit anti-hnRNP L, and anti-GAPDH antibodies were from Santa Cruz (Santa Cruz, CA). Mouse monoclonal anti-hnRNP L antibody was from Novus (Littleton, CO). Anti-HDAC1 and anti-β-actin antibodies were from Biovision (Mountain View, CA). Anti-c-Myc, anti-HA, goat anti-rabbit/mouse IgG (Alexa Fluor® 488 Conjugate), streptavidin-HRP, and anti-ubiquitin antibodies were from Cell Signalling Technology (Danvers, MA). Anti-DRBP76 antibody was from Biorbyt (Cambridge, UK). GST monoclonal antibody was from Thermo Scientific (West Palm Beach, FL). Anti-VHL antibody was from GeneTex (San Antonio, TX). Anti-hydroxyproline antibody was from Abcam (Cambridge, MA). Anti-rabbit IgG, anti-mouse IgG, and random-primer labeling kit were from GE healthcare (UK). Translation grade [^35^S]methionine was from NEN-Dupont (Boston, MA), α-[^32^P]CTP was from PerkinElmer (Boston, MA), and [^32^P]orthophosphoric acid was from MP Biomedicals (Solon, OH). Actinomycin-D, DMOG, and L-Mimosine were from Sigma (St. Louis, MO). *In vitro* ubiquitination assay kit and ubiquitin were from Biomol (Plymouth Meeting, PA) and Boston Biochem (Cambridge, MA), respectively.

### Cell Culture and Transfection

Human U937 monocytic cells (ATCC, Rockville, MD) were cultured in RPMI 1640 medium containing 10% heat-inactivated fetal bovine serum (FBS), 2 mM glutamine, and 100 U/ml of penicillin and streptomycin at 37°C and 5% CO_2_. PBM from healthy clinical donors were isolated by leukapheresis and countercurrent centrifugal elutriation under a Cleveland Clinic Institutional Review Board–approved protocol that adhered to American Association of Blood Bank guidelines. For preparation of cytosolic extracts, the cells were incubated for 1 h in medium containing 0.5% FBS and then with (or without) IFN-γ (500 units/ml) in presence of hypoxia (1% O_2_) for an additional 8 or 24 h. Cell lysates were prepared in Phosphosafe extraction buffer containing protease inhibitor cocktail. To knock down endogenous hnRNP L, DRBP76, hnRNP A2/B1, or VHL, U937 cells were transfected with appropriate concentration of (100–200 nM) gene-specific siRNA or a scrambled control siRNA using human monocyte nucleofactor kit. hnRNP L siRNAs containing 3 oligomers targeting the 3′UTR or ORF were from Origene. siRNA against DRBP76, hnRNP A2/B1, and VHL were from Santa Cruz.

### Plasmids, Site-Directed Mutagenesis, and Recombinant Protein Expression

The bacterial expression plasmid pRSET-hnRNP L was generated using pcDNA3-*hnRNPL-c-Myc* as template and cloned between *BamHI* and *EcoRI* restriction sites in the pRSET-A vector for expression and purification of His-tagged hnRNP L. *HNRNPL* ORF was subcloned into pGEX-4T-1 vector and the plasmid transformed into *E. coli* BL21(DE3) for expression and purification of GST-tagged hnRNP L. hnRNP L cDNA was subcloned into pcDNA3-*c-Myc* between *BamHI* and *EcoRI* restriction sites and expressed in human U937 cells as described [20]. The pcDNA3-based hnRNP L Tyr-to-Ala, -Asp, and -Phe mutants were prepared using GENEART Site-Directed Mutagenesis System (Invitrogen) according to the manufacturer's instructions. The mutation was confirmed by DNA sequencing. DRBP76 ORF was cloned into pET28-a vector between *NdeI* and *EcoRI* restriction sites. Expression of GST-tagged proteins was induced with 500 nM isopropyl-β-D-thiogalactopyranoside (IPTG) at 30°C for 6 h with 50 µg/ml ampicillin. Soluble protein was extracted and purified with B-PER GST purification kit (Thermo Fisher). His-tagged DRBP76 was generated *in vitro* using rabbit reticulocyte lysate *in vitro* translation system (Promega), and purified with MagneHis Protein Purification System (Promega). His-tagged wild-type hnRNP L and phospho-mimetic hnRNP L were expressed in *E. coli* BL21(DE3) with IPTG induction and in rabbit reticulocyte lysate *in vitro* translation system, respectively, and purified with Ni-NTA resin (Qiagen). Recombinant GST-hnRNP A2/B1 and hnRNP A2/B1 were from Novus Biologicals and Origene, respectively.

### Biotinylated RNA Affinity Purification and Mass Spectrometry

S100 extracts (4 mg) from U937 cells cultured in normoxia or hypoxia were pre-cleared by incubation for 30 min at 4°C with 2 µg 5-biotinylated, mutant antisense CARE-E RNA oligomer (5′-biotin-UCUGUGUGGGUGGGUGUAUGUAUGUAAAUA-3′), added to 200 µl of μMACs magnetic streptavidin microbeads for 10 min, and applied to μMACS separator. The cleared lysate was incubated with 2 µg of 5′-biotinylated, wild-type CARE-E RNA oligomer (5′-biotin-AGACACACCCACCCACAUACAUACAUUUAU-3′), and then with streptavidin microbeads and applied to μMACS separator as above. The column was rinsed with 100 µl protein equilibration buffer and twice with 100 µl of lysis buffer. The bound material was applied to the column and washed 4 times with 100 µl of lysis buffer to decrease nonspecific binding. 200 µl of buffer containing 300 mM NaCl was applied to the column to elute bound protein. The eluate was desalted and concentrated using Centrifugal Filter Unit (Microcon YM-3K, Millipore, Billerica, MA). Eluates were subjected to SDS-PAGE and Coomassie stain. Bands enriched only in hypoxia-treated sample were trypsinized and peptides mapped by capillary column LC-tandem MS (LTQ-linear ion trap MS system, ThermoFinnigan, San Jose, CA). The data were analyzed with Mascot using CID spectra to search the human reference sequence database. Matching spectra were verified by manual interpretation aided by additional searches using the Sequest and Blast.

### IP

Most IP experiments were done with Co-Immunoprecipitation kit (Pierce) following the manufacturer's instruction to eliminate antibody contamination of IP products. For some IP experiments, traditional method was used. Cells were lysed in Phospho-safe extraction buffer, and 500 µl of cell lysate was combined with 50 µl protein A/G agarose beads (50% bead slurry) and pre-cleared at 4°C for 60 min. The samples were centrifuged at 13,000 rpm for 10 min at 4°C and the supernatant added to 50 µl of protein A/G beads and 2 µg of antibody, and rotated for 4 h at 4°C. The beads were washed 5 times with 1 ml cold lysis buffer. Protein gel loading dye (100 µl) was added, and the samples boiled and loaded onto the gel. To avoid interference from IgG, rabbit-derived secondary antibody was used against mouse-derived primary antibody.

### *In Vitro* GST Pull-Down Assay

GST and GST-hnRNP L were generated from *E. coli* BL21(DE3) transformants containing pGEX-4T-1 and pGEX-4T-1-hnRNP L, respectively. Cells were sonicated and the supernatant collected after high-speed centrifugation. GST and GST-hnRNP L (1 µg of each) were incubated separately with glutathione-agarose beads for 30 min. After washing the agarose beads 4 times with 1 ml of PBS, 1 µg of recombinant DRBP76 and hnRNP A2/B1 were diluted in binding buffer (20 mM HEPES, pH 7.5, 200 mM KCl, 5 mM MgCl_2_, 0.2% bovine serum albumin, 10% glycerol, 0.1% Nonidet P-40, 1 mM phenylmethylsulfonyl fluoride, and complete protease inhibitor mixture), combined, and incubated at 4°C for 2 h. The agarose beads were washed 5 times with binding buffer (without bovine serum albumin and glycerol), and bound protein eluted by boiling in SDS loading buffer.

### Measurement of Protein Degradation

Cycloheximide (50 µg/ml) was added to 8×10^6^ U937 cells in 4 ml RPMI1640 medium. Cells were harvested and lysed. Immunoblot was done using anti-hnRNP L antibody and the band intensity quantified and normalized by the initial value at 0-h time point.

### *In Vitro* Ubiquitination Assay

*In vitro* reconstitution of hnRNP L ubiquitination was performed as described [55]. Purified His-tagged hnRNP L (0.5 µg) was preincubated with U937 cell lysate, and then incubated with a mixture of E1 and E2 enzymes, biotin-ubiquitin, and cell lysate as a source of hnRNP L E3 ligase. Recombinant hnRNP L was immunoprecipitated with anti-His tag antibody, and biotin-ubiquitin was detected by blotting with streptavidin-HRP.

### Metabolic Labeling by ^32^P-Orthophosphate

The metabolic labeling assay was performed as described previously [12]. U937 cells (8×10^6^ cells) in 4 ml RPMI 1640 medium were collected by centrifugation, re-suspended in phosphate-free medium, and metabolically labeled with a 4-h pulse of ^32^P-orthophosphate. The cells were collected by centrifugation and lysed with Phospho-safe extraction buffer containing protease inhibitor cocktail. hnRNP L was immunoprecipitated from lysates using mouse anti-hnRNP L antibody and protein A/G-agarose in cell lysis buffer. Proteins were resolved by 12% SDS-PAGE, and the gel was dried and applied to Phospho-screen for determination of radiolabeling.

### Analysis of HILDA Complex Constituents by Ultraviolet-Crosslinking

*In vitro* transcribed, ^32^P-labeled full-length HSR or truncated HSR RNA (20 fmol) was incubated for 30 min at 4°C with purified recombinant proteins (0.2 µg) in 20 µl of buffer containing 20 mM HEPES (pH 7.5), 5 mM MgCl_2_, 50 mM KCl, 1 mM DTT, protease inhibitor cocktail, 0.1% Triton X-100, 0.1 mg/ml yeast total tRNA, 40 U RNasin, and 10% glycerol. The mixture was crosslinked by 15 min exposure to ultraviolet light (1,800 J/cm^2^) on ice in a UV crosslinker. The protein-RNA complex was incubated with 1 µl of RNase A for 20 min at 25°C. Samples were denatured in SDS-PAGE buffer under reducing conditions, and complexes analyzed by 10% SDS-PAGE and autoradiography.

### RIP-RT-PCR

The RIP assay was performed as described previously [13]. Protein A/G beads (50 µl) were incubated with 500 µl of cell lysate (4 mg protein) for 1 h at 4°C with rotation to pre-clear. The cell lysate was centrifuged and the supernatant collected. Mouse anti-hnRNP L antibody (2 µg) was added (mouse pre-immune IgG was used as negative control) and the mixture incubated at 4°C overnight with rotation. Protein A/G beads (50 µl) were added and incubated at 4°C for 4 h. The beads were washed five times with 1 ml of lysis buffer with rotation at 4°C. Total immunoprecipitated RNA was extracted with Trizol. Total RNA from the lysate was extracted and used as a positive control for RT-PCR. Immunoprecipitated RNA (3 µl) and 1 µg of total RNA were used in reverse transcriptase reaction and subsequent PCR with Taq DNA polymerase. The PCR reaction (5 out of 20 µl) was visualized by 1.5% agarose gel. The primers for semi-quantitative RT-PCR were as follows: RT_βactin-f: 5′-ATGGATGATGATATCGCCGCG-3′; RT_βactin-r: 5′-CTAGAAGCATTTGCGGTGGAC-3′; RT_VEGF-f: 5′-ACAGAACGATCGATACAGAA-3′; RT_VEGF-r: 5′-AAAGATCATGCCAGAGTCTC-3′; RT_hnRNPL-f: 5′-GAGTCCCATCTGAGCAGGAA-3′; and RT_hnRNPL-r: 5′-CAATTTTATTGAAATGTGCC-3′.

### Isolation of Translationally Active and Inactive mRNA Pools by Sucrose Gradient Fractionation

Polysome profiling was done as described [13]. CHX (100 µg/ml) was added to cells for 15 min and then collected and washed two times with CHX-containing, ice-cold PBS. 10^7^ cells were suspended in 350 µl TMK lysis buffer and incubated on ice for 5 min. The lysates were centrifuged at 12,000 rpm for 10 min and the supernatants collected. RNase inhibitor (2 µl, 40 U/µl) and CHX (50 µl, 100 µg/µl) were added in 50 ml each of freshly prepared 10% and 50% sucrose gradient solutions just before use. Cytosolic lysates were loaded on the sucrose gradient and centrifuged at 29,000 rpm for 4 h, and 8 fractions of about 1 ml were collected and combined; light RNP, 40S, 60S, and 80S formed the translationally inactive pool, and heavy polysome fractions formed the translationally active pool. Total RNA was isolated from both combined fractions by extraction with Trizol reagent and purified by RNeasy minikit (Qiagen, Valencia, CA) following the manufacturer's procedure. The RNA was quantitated and purity determined by agarose formaldehyde gel, and used for real-time PCR analyses.

### *In Vitro* Translation

Capped, poly(A)-tailed template mRNAs was prepared using mMESSAGE mMACHINE SP6 and T7 kits (Ambion). Firefly-*Luc*-*VEGFA* GAIT element-poly(A) (200 ng) and Renilla-Luc (200 ng) reporter RNAs were incubated with U937 cytosolic lysates (500 ng of protein) from IFN-γ-treated U937 cells in the presence of 35 µl of wheat germ extract or rabbit reticulocyte lysate, and [^35^S]methionine. The translation reactions were performed for 90 min at 30°C and resolved by SDS-PAGE (10% polyacrylamide) and visualized by phosphorimaging. In some experiments, the FLuc and RLuc activity was measured by chemiluminescence using luminator.

### Dual Luciferase Reporter Assay

U937 cells were transiently transfected with 5 µg of wild-type or mutant pCD-FLuc-VEGFA HSR using human monocyte nucleofactor kit. RLuc-expressing vector pRL-SV40 (1 µg) was co-transfected for normalizing transfection efficiency. After 12 h, transfected cells were incubated with IFN-γ under Nmx. or Hpx. for up to 24 h, lysed, and lysate luciferase activities were measured using a dual luciferase assay kit (Promega). The primers for semiquantitative RT-PCR of FLuc were as follows: RT_FLuc-f: 5′-GCCTGAAGTCTCTGATTAAGT-3′; RT_FLuc-r: 5′-ACACCTGCGTCGAAGT-3′; RT-RLuc-f: 5′-TGATTCAGAAAAACATGCAG-3′; RT-RLuc-r: 5′-ATATTTGTAATGATCAAGTA-3′.

### Immunofluorescence

Immunostaining of hnRNP L was as described [23]. U937 cells (10^6^ cells/ml) in 12-well plates with glass cover slip at the bottom were incubated in hypoxia or normoxia for 24 h. Cells were centrifuged for 5 min at 2,500 rpm and washed twice with PBS and then with 4% paraformaldehyde fixing solution for 20 min. Cells were washed twice with PBS, and incubated with rabbit anti-hnRNP L polyclonal antibody (Santa Cruz, 1∶40) in blocking solution (2% BSA, 0.1% Triton X100 in PBS) at room temperature for 2 h. Cells were washed twice with PBS and centrifuged at 1,500 rpm for 5 min. Alexa Fluor 488 goat anti-rabbit secondary antibody (Invitrogen) was added (1∶50) with phalloidin (1∶50) in blocking solution for 1 h. Cells were washed with PBS three times. DAPI dye was mixed in the mounting solution and the slides imaged.

## Supporting Information

Figure S1 **Hypoxia-induced suppression of GAIT-mediated translational silencing of VEGF-A.** U937 cells (left) and PBM (right) were treated with IFN-γ in Nmx. or Hpx., and VEGF-A protein and *VEGFA* mRNA in lysates were determined by immunoblot and RT-PCR, respectively; GAPDH and *β-actin* were probed as controls.
(EPS)

Figure S2 **Co-requirement of HILDA complex constituents for binding to** ***VEGFA*** **mRNA** ***in vivo*** **.** U937 cells were transfected with hnRNP A2/B1-, DRBP76-, and hnRNP L-specific (or scrambled) siRNA, and lysates immunoprecipitated with anti-DRBP76 and anti-hnRNP A2/B1 antibody, respectively. Extracted RNA was subjected to RT-PCR using primers specific for *VEGFA* or *β-actin* mRNA.
(EPS)

Figure S3 **HSR sequence, mutagenesis strategy and predicted secondary structures, and** ***VEGFA*** **mRNA half-life.** (A) The position and sequence of CARE, GAIT, and AUSL elements in HSR region. CARE element is from C^337^ to U^357^. GAIT element is from U^358^ to A^386^. AUSL element is from A^364^ to U^426^. (B) Schematic of HSR mutagenesis strategy for mapping the minimal binding element for DRBP76. The mutation sequence of M1, M2, M3, and M4 are shown in details in the text. (C) Schematic of HSR mutagenesis strategy for determining the spacer limitation between CARE and GAIT elements in active VEGF-A RNA switch. 5, 10, 15, 20, and 25 of (C) are inserted between U^357^ and U^358^ as shown. (D) Measurement of the half-life of *VEGF-A* mRNA in U937 cells in the absence of DRBP76. U937 cells were transfected with scrambled siRNA or DRBP76-specific siRNA in hypoxic condition. After recovery for 24 h, the cells were treated with 2 µg/ml Actinomycin D up to 4 h. Total mRNA were extracted from the cells by Trizol reagent, and *VEGF-A* mRNA level was measured by qRT-PCR.
(EPS)

Figure S4 **Polysome profiles.** A_260_ absorbance profiles are shown following sucrose gradient fractionation; translationally inactive and active pools are indicated as non-polysome (black bar) and polysome (gray bar) fractions, respectively.
(EPS)

Figure S5 **Steady-state level of hnRNP L mRNA was determined by Northern blot.** U937 cells treated with IFN-γ under normoxia or hypoxia for up to 24 h. *hnRNP L* and *β-actin* mRNA were determined by Northern analysis using gene-specific probes.
(EPS)

Figure S6 **Protein loading controls for** **Figure 4B and 4C** **.** (A) Loading control for **Figure 4B**. (B) Loading control for Figure 4C. Total proteins were visualized with Coomassie blue stain.
(EPS)

Figure S7 **pVHL does not affect assembly of the HILDA complex in hypoxia.** Overexpression of pVHL does not affect the expression of HILDA component proteins or disturb the assembly of HILDA complex. U937 cells were transfected with plasmid encoding HA-VHL and then incubated in hypoxia for up to 24 h. Lysates were immunoprecipitated with anti-hnRNP L antibody and then immunoblotted with antibodies against HILDA constituents. Western blots were performed with antibodies against all HILDA proteins as input control.
(EPS)

Figure S8 ***In vitro*** **reconstitution of hnRNP L ubiquitination.** His-tagged hnRNP L was incubated with biotin-ubiquitin and a reconstituted ubiquitination system containing recombinant E1 and E2 and lysate from cells treated with IFN-γ for 8 h in Nmx. or Hpx. (or cells treated with IFN-γ for 0 h as negative control) to provide E3 ligase activity.
(EPS)

Figure S9 **Hypoxia induces cytoplasmic localization of hnRNP L in primary human PBM-derived macrophages.** Primary human PBM was incubated with 100 ng/ml M-CSF for 7 d and treated with IFN-γ for 24 h under normoxia or hypoxia. The differentiated macrophages were immunostained using mouse anti-hnRNP L monoclonal antibody (Santa Cruz, 1∶80) and goat anti-mouse IgG (Alexa Fluor 488 Conjugate, 1∶50). Cell nuclei were stained with DAPI.
(EPS)

Figure S10 **Mass spectrometric determination of candidate sites for hypoxia-dependent phosphorylation of hnRNP L.** hnRNP L was immunoprecipitated from cytosolic lysates of hypoxia-treated U937 cells, and peptides detected by mass spectrometric analysis following digest with chymotrypsin (blue), trypsin (red), or both (green). Candidate Tyr residues in peptides not observed in any digest (black) are indicated (underline).
(EPS)

Figure S11 **Cellular localization of phospho-dead hnRNP L in cells treated with hypoxia plus IFN-γ.** c-Myc-tagged, wild-type (WT), and phospho-dead (Tyr-to-Ala, Y359A, left or Tyr-to-Phe, Y359F, right) mutant hnRNP L were transiently transfected into U937 cells, treated with IFN-γ in Hpx., and were determined in cytoplasmic and nuclear fractions by immunoblot analysis with anti-c-Myc, -HDAC1, and -tubulin antibodies.
(EPS)

Figure S12 **Condition-dependent posttranslational modification of hnRNP L.** U937 cells were treated with IFN-γ in normoxia in the presence of MG132, in Hpx. alone, and IFN-γ in Hpx. Immunoprecipitated hnRNP L was blotted with anti-P-Tyr and anti-hydroxyproline antibodies.
(EPS)

Figure S13 **Knockdown of DRBP76 does not affect hnRNP L stability.** U937 cells were transfected with DRBP76-specific (or scrambled) siRNA. After recovery, cells were treated with IFN-γ and Hpx. for up to 24 h. Lysates were immunoblotted with anti-DRBP76 and -hnRNP L antibodies.
(EPS)

Figure S14 **Hydroxylase inhibitors prevent prolyl hydroxylation and degradation of hnRNP L.** U937 cells were treated with 500 µM DMOG or 100 µM L-Mimosine in the presence of MG132 in Nmx. for 24 h, and hnRNP L was immunoprecipitated with specific antibody and immunoblotted with anti-hydroxyproline antibody. In the absence of MG132 treatment, Western blots were done with anti-hnRNP L and anti-GAPDH antibodies.
(EPS)

Figure S15 **Reconstitution of HILDA complex** ***in vitro*** **.** DRBP76 and hnRNP A2/B1 were pre-incubated (5 pmol each) for 0.5 h on ice. Phospho-mimetic (Y359D) or wild-type hnRNP L (5 pmol) were added in the presence of 5 pmol of HSR RNA or nonspecific RNA (NS, 109-nt RNA was generated by *in vitro* transcription of the EPRS coding region polyadenylation cassette [13]), and incubated for 1 h on ice. Samples were subjected to IP with anti-DRBP76 antibody and immunoblot with anti-hnRNP A2/B1 antibody. Input levels of each protein were determined by immunoblots.
(EPS)

Table S1 **Mass spectrometric analysis of HILDA complex constituents.** Peptides, coverage, and Mascot scores by mass spectrometric analysis of hnRNP L, DRBP76, and hnRNPA2/B1.
(DOC)

## Abbreviations

AUSL: AU-rich stem loop
CARE: CA-rich element
DMOG: dimethyloxalylglycine
DRBP76: double-stranded RNA binding protein 76
EPRS: glutamyl-prolyl-tRNA synthetase
GAPDH: glyceraldehyde 3-phosphate dehydrogenase
HIF: hypoxia-inducible factor
HILDA: hypoxia-inducible hnRNP L–DRBP76–hnRNP A2/B1
hnRNP: heteronuclear ribonucleoprotein
Hpx.: hypoxia
HSR: hypoxia stability region
IFN: interferon
Nmx: normoxia
PBM: peripheral blood monocytes
VEGF: vascular endothelial growth factor
VHL: von Hippel-Lindau
