# Supplementary material for: The HILDA Complex Coordinates a Conditional Switch in the 3′-Untranslated Region of the VEGFA mRNA
Source: PLoS Biol. 2013 Aug 20;11(8):e1001635. doi: 10.1371/journal.pbio.1001635 (PMC3747992; doi:10.1371/journal.pbio.1001635)
Supplement: Table S1 — Mass spectrometric analysis of HILDA complex constituents. Peptides, coverage, and Mascot scores by mass spectrometric analysis of hnRNP L, DRBP76, and hnRNPA2/B1. (DOC) [file pbio.1001635.s016.doc]

**Table S1 Mass spectrometric analysis of HILDA complex constituents.**

| Protein | Molecular weight (kDa) | Peptide coverage | Mascot score | Peptides detected |
| --- | --- | --- | --- | --- |
| hnRNP L | 64 | 16% | 383 | T^98^PASPVVHIR^107^  I^179^SRPGDSDDSR^189^  I^265^EYAKPTR^272^  M^345^GPPVGGHR^353^  V^399^FNVFCLYGNVEK^411^  A^435^ITHLNNNFMFGQK^448^  F^485^STPEQAAK^493^  N^569^PNGPYPYTLKLCFSTAQHAS^589^ |
| DRBP76 | 95 | 10% | 432 | V^183^LAGETLSVNDPPDVLDR^200^  C^203^LAALASLR^211^  ^225^SCVIVIR^231^  E^298^ATDAIGHLDR^308^  ^312^EDITQSAQHALRLAAFGQLHK^332^  A^397^EPPQAMNALMR^408^  ^455^LHVAVK^460^  ^604^APVPVRGGPK^613^ |
| hnRNP A2/B1 | 37 | 18% | 342 | T^4^LETVPLER^12^  ^113^KLFVGGIK^120^  ^138^IDTIEIITDR^147^  ^154^GFGFVTFDDHDPVDK^168^  ^204^GGNFGFGDSRGGGGNFGPGPGSNFR^228^ |
